# The shape of gene expression distributions matter: how incorporating distribution shape improves the interpretation of cancer transcriptomic data

**DOI:** 10.1186/s12859-020-03892-w

**Published:** 2020-12-28

**Authors:** Laurence de Torrenté, Samuel Zimmerman, Masako Suzuki, Maximilian Christopeit, John M. Greally, Jessica C. Mar

**Affiliations:** 1 Department of Systems and Computational Biology, Albert Einstein College of Medicine, Bronx, NY 10461 USA; 2 Center for Epigenomics and Department of Genetics, Albert Einstein College of Medicine, Bronx, NY 10461 USA; 3 Internal Medicine II, Hematology, Oncology, Clinical Immunology and Rheumatology, University Hospital Tuebingen, Otfried-Mueller-Strasse 10, 72076 Tuebingen, Germany; 4 Department of Epidemiology and Population Health, Albert Einstein College of Medicine, Bronx, NY 10461 USA; 5 Australian Institute for Bioengineering and Nanotechnology, The University of Queensland, Brisbane, QLD 4072 Australia

**Keywords:** Gene expression, Multi-modality, Non-normal distribution, Survival analysis, Cancer genomics

## Abstract

**Background:**

In genomics, we often assume that continuous data, such as gene expression, follow a specific kind of distribution. However we rarely stop to question the validity of this assumption, or consider how broadly applicable it may be to all genes that are in the transcriptome. Our study investigated the prevalence of a range of gene expression distributions in three different tumor types from the Cancer Genome Atlas (TCGA).

**Results:**

Surprisingly, the expression of less than 50% of all genes was Normally-distributed, with other distributions including Gamma, Bimodal, Cauchy, and Lognormal also represented. Most of the distribution categories contained genes that were significantly enriched for unique biological processes. Different assumptions based on the shape of the expression profile were used to identify genes that could discriminate between patients with good versus poor survival. The prognostic marker genes that were identified when the shape of the distribution was accounted for reflected functional insights into cancer biology that were not observed when standard assumptions were applied. We showed that when multiple types of distributions were permitted, i.e. the shape of the expression profile was used, the statistical classifiers had greater predictive accuracy for determining the prognosis of a patient versus those that assumed only one type of gene expression distribution.

**Conclusions:**

Our results highlight the value of studying a gene’s distribution shape to model heterogeneity of transcriptomic data and the impact on using analyses that permit more than one type of gene expression distribution. These insights would have been overlooked when using standard approaches that assume all genes follow the same type of distribution in a patient cohort.

## Background

A fundamental tenet of applied statistics states that under certain conditions, data can be modeled by a probability distribution like a Normal or a Poisson. If appropriately applied, then this assumption is powerful because it allows for well-established statistical methods to be used to answer questions about the data. The most common statistical methods, such as the t-test and ANOVA models, are all predicated on this assumption that the data follows a Normal distribution. As we begin to learn more about the diversity of gene expression in human populations, we call into question the relevance of assuming that the transcriptome can be uniformly modeled by just one distribution. At the heart of our study is a straightforward question—how prevalent are genes with non-Normal expression, and what new information for understanding transcriptional regulation can we learn from them? We raise this question not to invalidate previous findings that have used assumptions of Normality, but instead to draw attention to genes that may have otherwise been overlooked and the insights that they bring, especially in the context of disease processes. Using the Cancer Genome Atlas (TCGA) as a platform to investigate this question, we show that more than half of genes in the cancer transcriptome are non-Normally distributed for multiple tumor types. Most significantly, we show that accounting for the distribution shape improved the accuracy of patient survival time predictions. Our study demonstrates that the assumption of Normality did not apply uniformly to all genes in the cancer transcriptome. Importantly, incorporating assumptions based on multiple distribution categories into the analysis of gene expression revealed information for understanding the transcriptional control of cancer that would have been missed using standard approaches.

**Fig. 1.**
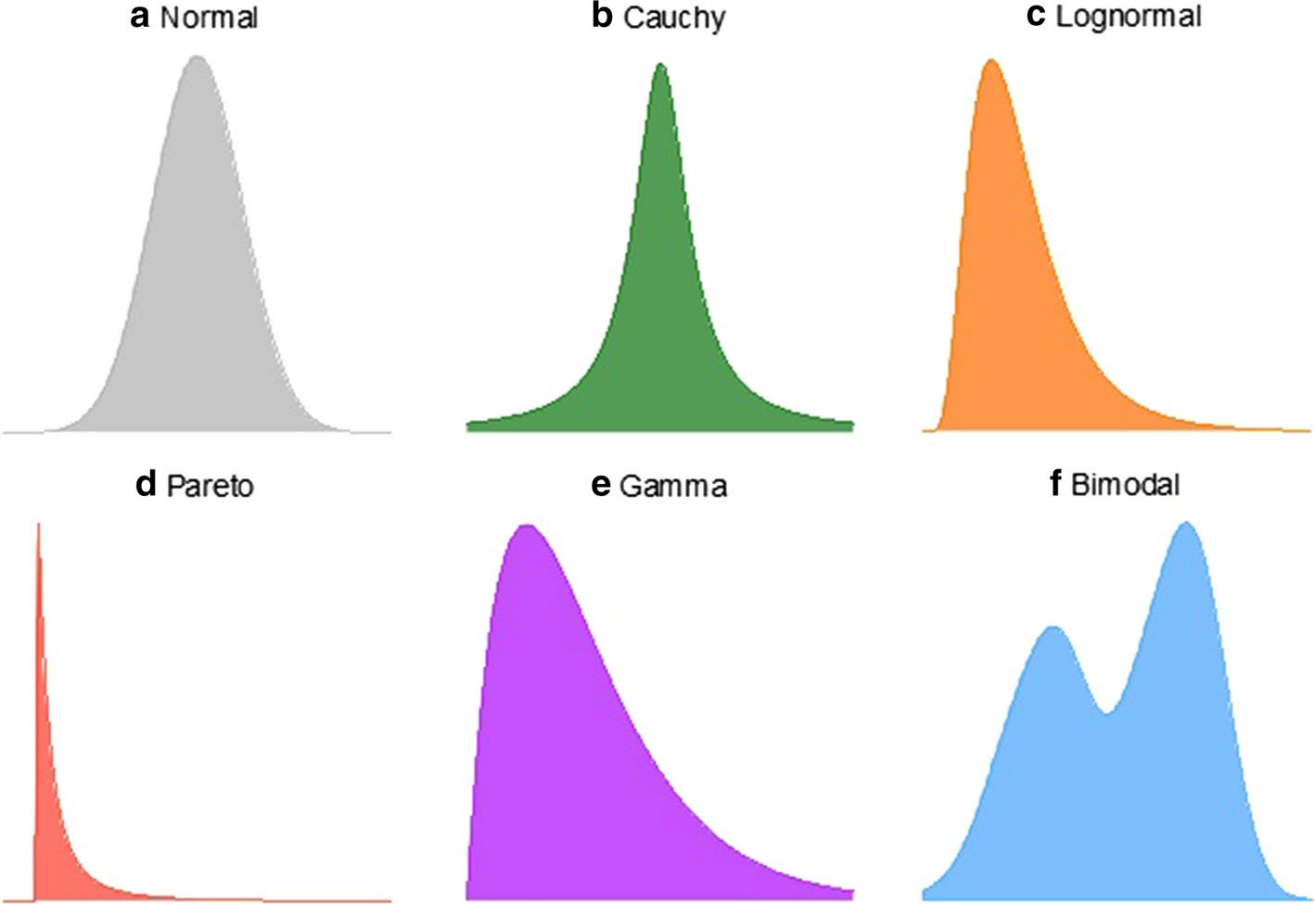
Panel of six statistical distributions that capture a diversity of different probability density shapes. **a** Normal, **b** Cauchy, **c** Lognormal, **d** Pareto, **e** Gamma, **f** Bimodal

To investigate the prevalence of Normally-distributed genes in the transcriptome, we assembled a panel of six statistical distributions. Each of these distributions has its own set of properties and collectively capture a diversity of density shapes (Fig. 1). We included two symmetric distributions, the Normal and Cauchy distributions, where the latter has heavy tails and is more peaked than the Normal distribution. The Lognormal, Pareto, and Gamma distributions all have skewed, asymmetric shapes. The Lognormal is an asymmetric distribution but on a log scale, so Normality assumptions are still applicable for this distribution. In contrast, the Pareto is a heavy-tailed distribution and the most skewed in our distribution panel. The Gamma is a distribution whose values can span more or less skewness depending on the parameters, and overall its shape is not as extreme as the Pareto. The Bimodal distribution models an alternative kind of heterogeneity where two distinct sub-groups exist in the data. We applied this panel to three different TCGA data sets, the acute myeloid leukemia (AML) [1], ovarian cancer (OV) [2], and Glioblastoma multiforme (GBM) [3] patient cohorts.

## Results

### Over 50% of the cancer transcriptome does not follow a Normal distribution.

**Fig. 2:**
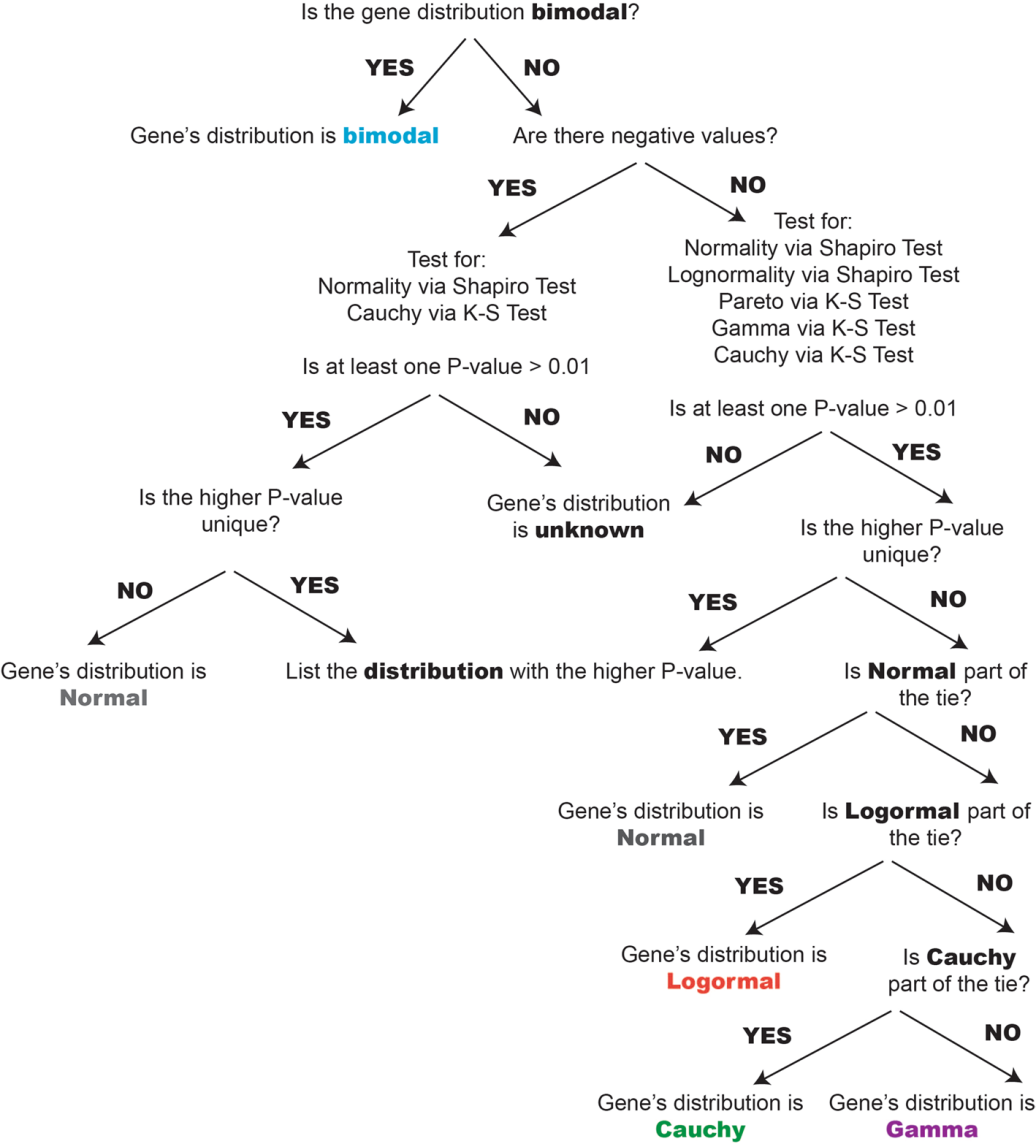
Pipeline to classify genes according to their distribution shape. If a gene is classified as bimodal, it is removed from the list and the algorithm continues on the remaining genes. The category sets are mutually exclusive. Genes falling in neither category are classified as unknown and removed from further analysis

**Fig. 3:**
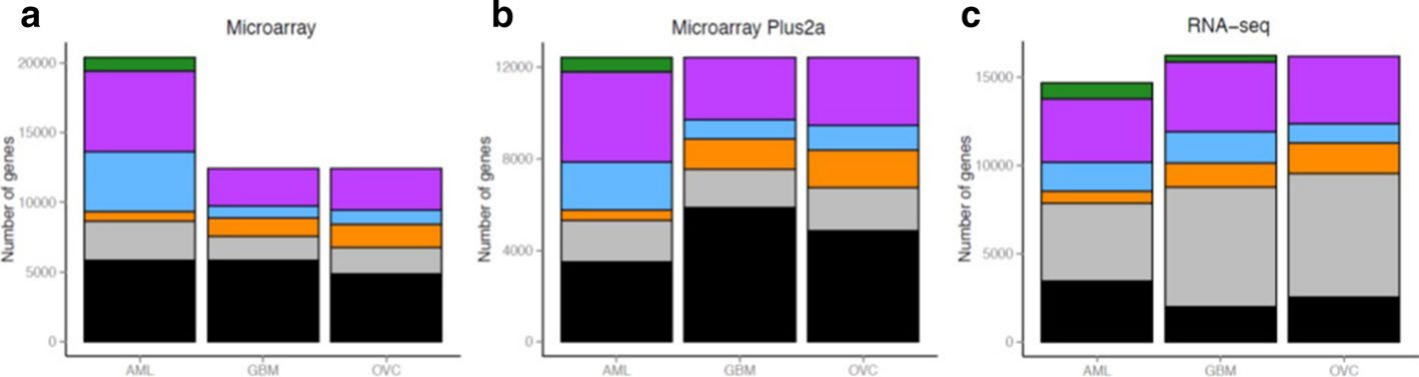
Representation of genes in each distribution category for the microarray, microarray Plus2a, RNA-seq data. **a** Number of genes classified in each distribution for the three microarray datasets. **b** Number of genes classified in each distribution when exclusively looking at the genes in the Plus2a platform **c** Number of genes classified in each distribution for the three RNA-seq datasets. Colors correspond to different distributions; Gamma (purple), bimodal (bimodal), Cauchy (green), Lognormal (orange), Normal (gray), unknown (black). Note that no genes in the Pareto distribution were identified for any of the datasets

Each gene was classified based on the proximity of its gene expression distribution to one of the six probability density distributions described in Fig. 2. The classification scheme was designed to first evaluate the statistical likelihood of whether a gene’s expression profile matched a Bimodal distribution, and if this was not the case, to then assess whether any of the remaining five unimodal distributions were a more appropriate fit (see Methods for a description of the classification scheme). If an adequate fit could not be determined from these six options, the gene was discarded from further analysis. Under this classification scheme, the Normal distribution captured 13 to 15% of genes in the three microarray cancer datasets in this study (13.73% for AML, 13.52% for GBM, 15.13% for OV, Fig. 3a) and less than 45% of genes in the RNA-seq datasets for the same tumor types (30.29% for AML, 41.8% for GBM, 43.18% for OV, Fig. 3c). The Gamma distribution was the largest non-Normal category of genes for both microarray and RNA-seq datasets (representing 21–32%). Gene counts for all distributions are listed in Additional file 1: Table S1. Since different microarray platforms were used to generate the data, we investigated whether this affected any of the distribution counts observed (Fig. 3a). Because the two Affymetrix microarray platforms that were used, U133A and U133 Plus 2.0, share a set of genes in common, the presence of a platform-specific effect could be tested. When focusing only on the set of genes that were common across all array platforms, it could be seen that the proportion of genes assigned to each of the distributions remained the same, indicating that this effect is more likely to be biological rather than technical (Fig. 3b).

### Incorporating assumptions that permit more than one distribution type identifies different sets of genes that discriminate between good versus poor patient survival outcomes

**Fig. 4:**
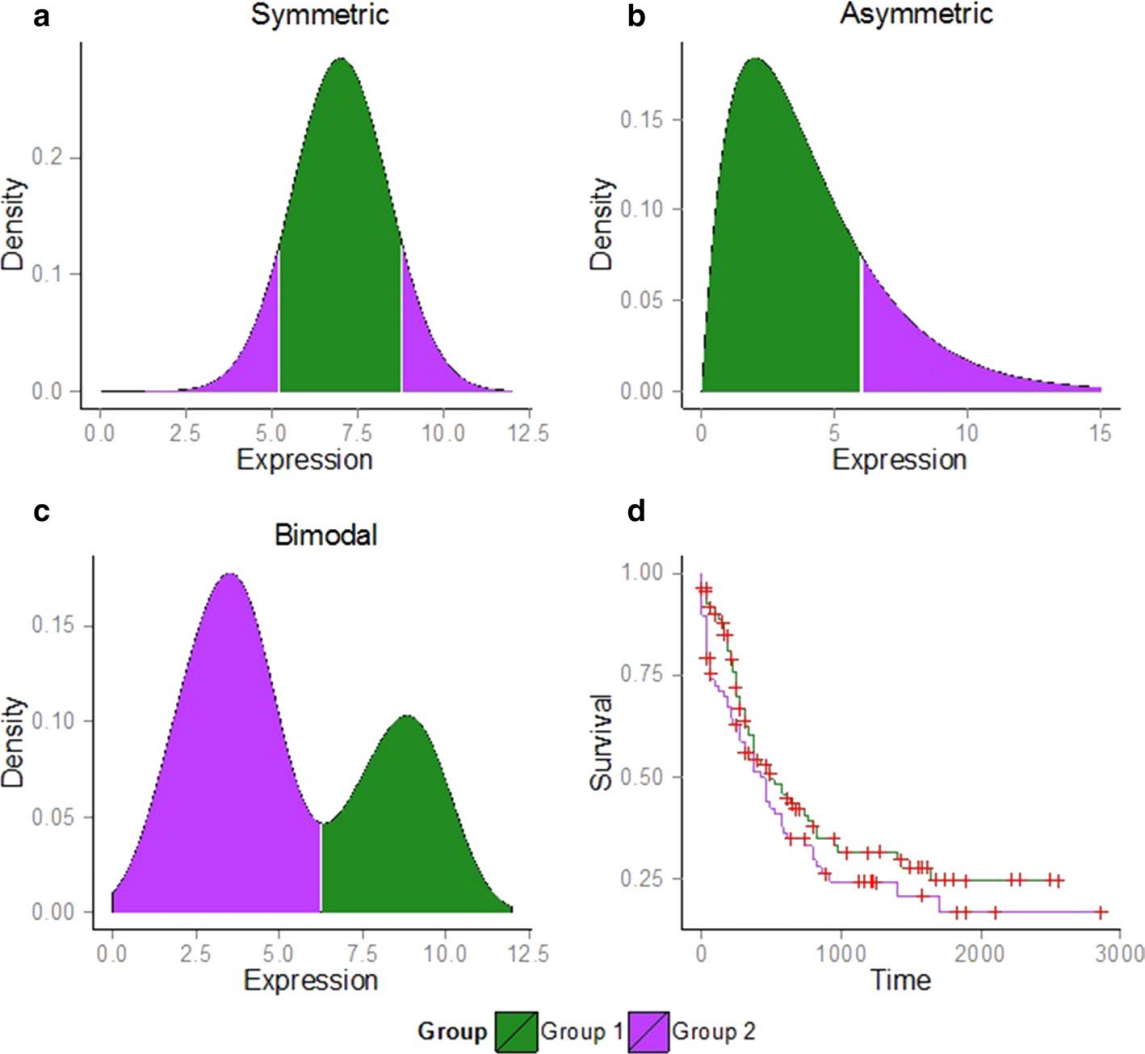
Decision rules for identifying extreme and non-extreme patient groups that take into account the specific shape of the gene expression distribution. **a** For symmetric distributions, the extreme patient group is represented by the samples falling in either the upper or lower percentile of the distribution as shown by the purple tails (in our analysis, the tenth percentile is used). The non-extreme patient group corresponds to the samples falling between these two percentile cut-offs as shown by the green region. **b.** For asymmetric distributions, the extreme patient group corresponds to samples only in the first or last percentile depending on the shape of the asymmetry, as shown by the one-sided purple tail. The remaining region of the distribution represents the non-extreme patient group. **c** For bimodal distributions, the split is determined by a clustering algorithm applied to the expression data to identify which patients belong to one group (mode) versus another. For genes in the bimodal expression category, the definition of extreme and non-extreme patient groups is not relevant, and instead we identify two patient groups for comparison, as shown by the purple and green regions. **d** Theoretical example of two survival curves constructed for patients in Groups 1 and 2 as defined in a, b or c

In a cancer patient cohort, gene expression is commonly used to identify genes that can discriminate between patients with good versus poor survival. Typically, the methods employed for this purpose assume that all genes follow the same underlying distribution. This may be limited because the task of identifying meaningful sub-groups is affected by the shape of a gene’s expression profile in the patient cohort. For example, if a gene is assumed to have Normally-distributed data (or any symmetric distribution), we usually compare the patients that have expression of a gene in the extreme tails of the distribution, against those with gene expression in the non-tail region (Fig. 4a). For symmetrically-distributed data, like the Normal distribution, this is a sensible sub-grouping to adopt. However, for non-Normal distributions, such as those with asymmetry or are bimodal, it is clear that an alternative, more intuitive choice of sub-grouping is available (Fig. 4b, c) for contrasting survival curves (Fig. 4d).

We term those genes whose expression distinguishes significant differences in patient survival time as prognostic marker genes. We found that when the distribution shape is taken into account, a set of prognostic marker genes was identified (log-rank test, *p* value < 0.05, see Additional file 1: Table S2). The intersection between the genes that were found when the distribution shape information was used, and those when the assumption of symmetry was applied uniformly (i.e. assumption of only one distribution), was minimal for the three cancers (for the non-symmetric genes, the overlap was eight genes for AML, and zero for GBM and OV). This small overlap indicates that a different set of prognostic markers are identified, depending on whether the distribution shape is factored in, or a single distribution type is assumed.

The second largest type of non-Normal distribution represented amongst the genes that were identified based on the distribution shape information was the Bimodal distribution. This result highlights the existence of Bimodally-expressed genes that have distinct modes corresponding to statistically significant differences in survival time. Genes that were found to be significant in patient survival time using the shape of the expression distribution are listed in Additional file 1: Table S3 (summary of survival times listed in Additional file 1: Table S4). To determine the degree of robustness of our result, we also looked at how many genes were significant in survival time when patients were randomly assigned to the two groups (corresponding to the green and purple regions in Fig. 4). No genes were significant under this assumption, indicating that the genes found using the distribution shape information were unlikely to be detected purely by chance (Additional file 1: Table S2).

### Identifying prognostic marker genes using the expression distribution shape information provides functional insights into cancer biology that were not found using standard symmetric assumptions

For AML, functional terms and biological pathways from MSigDB were significantly over-represented in the non-Normal prognostic marker genes, and not in the set of Normally-expressed prognostic marker genes, indicating that these gene sets correspond to different pathways (Additional file 1: Table S5–S7). The non-Normal prognostic marker genes for AML were enriched for the KEGG inositol phosphate metabolism pathway. Previous studies have demonstrated a link between this pathway and cancer, where common germline variation in this pathway has been shown to serve as a susceptibility factor [4, 5]. Another KEGG pathway that was enriched was related to Fc gamma R-mediated phagocytosis, a pathway that has previously been shown to be upregulated in HL-60 cells, which is a leukemia cell line [6]. The non-Normal prognostic marker genes in AML were also enriched for an oncogenic signature based on human leukemia cells from a HOXA9 knockdown (Additional file 1: Table S5). We investigated whether any of our non-Normal genes that were identified using the distribution shape information had been detected in seven previous studies of AML gene expression [7–12]. Of all the seven signatures tested, we observed at most two genes out of 561 in the signature, suggesting that the non-Normal genes we have identified may be prognostic markers of AML that have largely been missed by existing analyses (Additional file 1: Table S8).

For OV, the pathways that were exclusively over-represented in the non-Normally expressed genes were enriched for the MicroRNA biogenesis REACTOME pathway. It has been shown that gene sets related to RNase III DROSHA and DICER1 were decreased in ovarian cancer [13]. The non-Normal prognostic marker genes were also enriched in genes defining epithelial-mesenchymal transition which is a critical step for cancer cell invasion and metastasis [14] (Additional file 1: Table S7).

For GBM, the non-Normal prognostic marker genes were enriched for the immune system and neuronal system pathways from REACTOME, and more broadly for gene sets involved in the regulation of cellular and biological processes (Additional file 1: Table S6). Overall, for this particular tumor type, the pathways over-represented in the list of non-Normal prognostic marker genes were less specialized and with less of a clear link to cancer compared to the other two tumor types.

### Incorporating the shape of the gene expression distribution improved the performance of a classifier’s ability to predict survival of individual patients in different types of cancers

Prognostic marker genes can be combined to construct a gene expression-based classifier to predict the survival time of new patients. We investigated whether leveraging information about the expression distribution shape resulted in more accurate predictions of patient survival time compared to classifiers that assumed all genes followed a symmetric distribution. To compare the performance of these two sets of predictors, we used a non-parametric classification method, a random survival forest, on genes that were selected based on whether they were significantly different in gene expression under the two sets of assumptions.

Because of its ability to distinguish relevant features from irrelevant ones, the random survival forest method [15] is well-suited for high-dimensional problems. It is an efficient computational method that can handle non-linear or complex higher-order interaction effects. Under the algorithm framework, a tree represents a graphical construct that describes the hierarchical relationship between genes. Individual genes are prioritized in the hierarchy based on how well their expression profiles are able to discriminate between patient survival times. A collection of trees, termed a forest, is grown by the algorithm using independent bootstrapping of the original data set. For each tumor type, we designated two-thirds of the data for classifier training, and the random survival forest method was applied to this training set using 1000 bootstraps. The remaining one-third of the data was used for testing the accuracy of the classifier by predicting the survival status of each patient that the classifier had not yet seen.

**Fig. 5:**
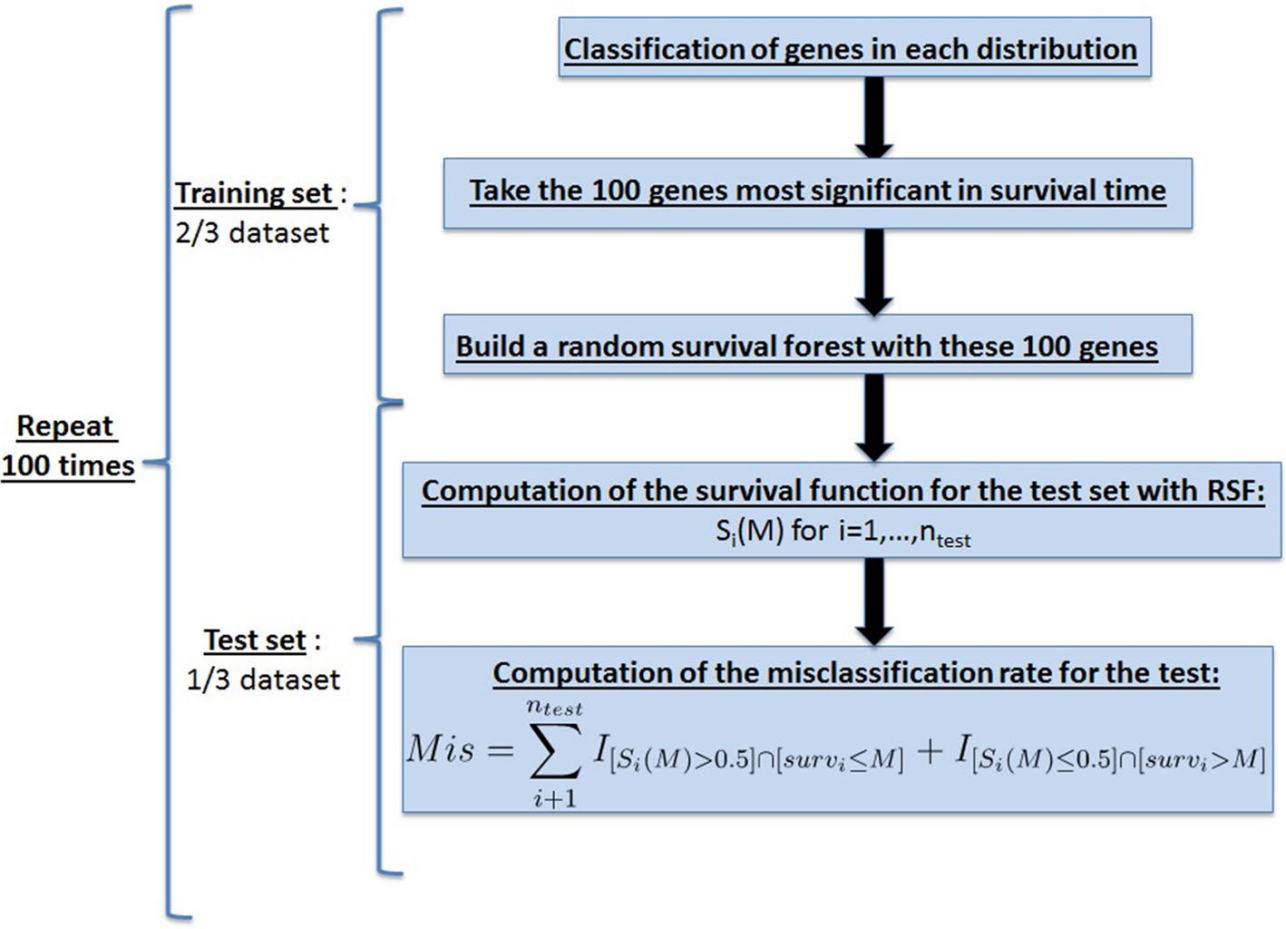
Pipeline to test the performance of the shape-based assumption in patient survival prediction. This pipeline is used once for each assumption: shape, symmetric and random where the difference occurs in step 2. For the shape assumption, we will separate the samples using the shape (Fig. 4a–c) and then compute the *p* value with a log-rank test. For the symmetric assumption, we will separate the samples into two groups using the same splitting rule for all the genes (Fig. 4a) and then compute the *p* value with a log-rank test. For the random assumption, the 100 genes are just chosen at random from the dataset and then compute the *p* value with a log-rank test

Classifiers were constructed under the two assumptions that differed in how the patient subgroups were identified. For the first assumption, the shape of the gene expression distribution was taken into consideration so that the subgroups were defined as in Fig. 4b. For the second assumption, all genes were assumed to have symmetric expression distributions (Fig. 4a). To test the robustness of the results, a third set of classifiers was also constructed based on a random selection of genes that was equal in size to the number of genes obtained under the shape-based assumption. To ensure that our results were not biased toward a specific combination of patients in the training and test data sets, we randomly divided the data into training and test sets 100 times with a 2/3 split of the data set for the training set and 1/3 for the test set. The classification of genes in each distribution was restricted to the training set only. The classification procedure was repeated under the three sets of assumptions for the three different tumor types for each of the 100 unique training/test data sets. Performance of the three classifiers was assessed based on misclassification rates observed for the 100 repeats of each tumor type using the test data set. A prediction was considered misclassified if a patient was predicted to have good survival when in actual fact the patient’s survival status was poor, or vice versa (Fig. 5).

**Fig. 6:**
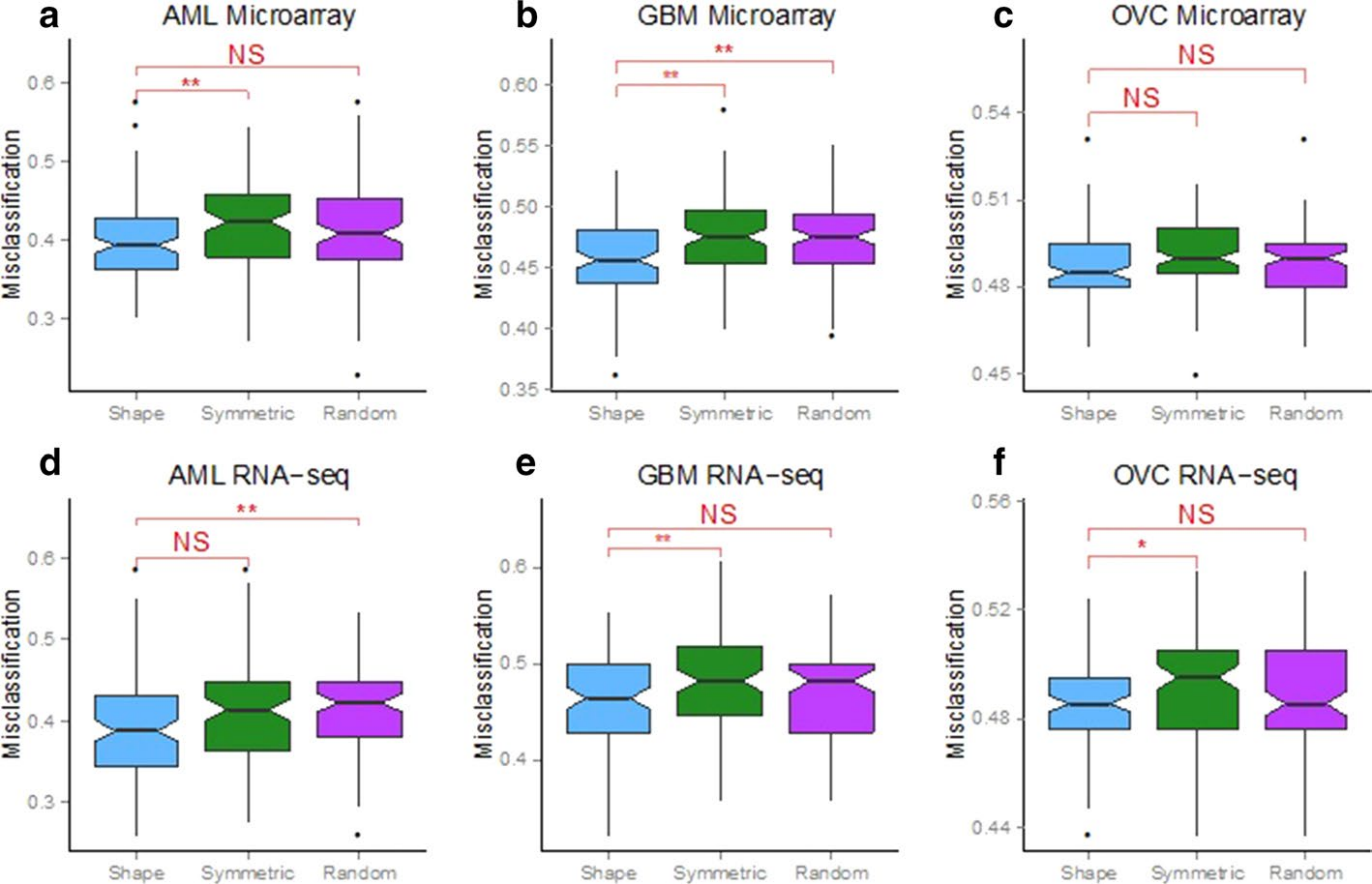
Comparing prediction accuracy using classifiers that incorporate the expression shape versus assuming a symmetric distribution for all genes. We used random survival forests to predict the prognosis of patients and tested the performance of classifiers derived three ways; first, incorporating information from the distribution shape, second, assuming symmetry for all genes, and third, for a random set of genes. Classifiers were trained on 2/3 of the data, tested on 1/3, and repeated 100 times **a** AML Microarray, **b** GBM Microarray, **c** OV Microarray, **d** AML RNA-seq, **e** GBM RNA-seq, **f** OV RNA-seq. Stars indicate datasets where the shape-based approach produced lower misclassification rates that were statistically significant (Wilcoxon test, * = *p* value < 0.05, ** = *p* value < 0.01, NS = Not Significant). The notch in each boxplot displays a confidence interval based on median misclassification rate ± 1.58 × IQR/√n where n = 100, notches that do not overlap reflect statistically significant comparisons

Classifiers derived under the shape-based assumption surpassed the performance of the symmetric-based ones for the microarray and RNA-seq datasets of the three cancer types, AML, GBM and OV (Fig. 6). Performance of the classifiers can also be assessed by counting how many times the shape-based classifier outperformed the symmetric-based classifier in the 100 repeats performed. Using this metric, the shape-based classifier performed as well or better than the symmetric-based one in at least 60 of the 100 repeats (Table 1). In AML, 63 out of 100 times for microarray and 60 out of 100 times, the shape-based classifier had an equal or better misclassification rate than the symmetric one. The two classifiers had even better performance for GBM and OV with a 66 and 69 out of 100 times for microarray and a 71 and 70 out of 100 times for RNA-seq, respectively. In summary, building the shape of the gene expression distribution results into classifiers that predict a patient’s survival time increased performance.

**Table 1:** Number of times where the shape assumption is better than the symmetric one for Microarray and RNA-seq datasets

	AML Microarray	GBM Microarray	OV Microarray	AML RNA-seq	GBM RNA-seq	OV RNA-seq
Misclassification rate of shape assumption ≤ misclassification rate of symmetric assumption	63 (7)	66 (2)	69 (16)	60 (7)	71 (13)	70 (14)

The number in parenthesis represents the number of equality between both misclassification rates

### Box-Cox transformations did not alter the number of Normally-distributed genes in RNA-sequencing data

**Fig. 7:**
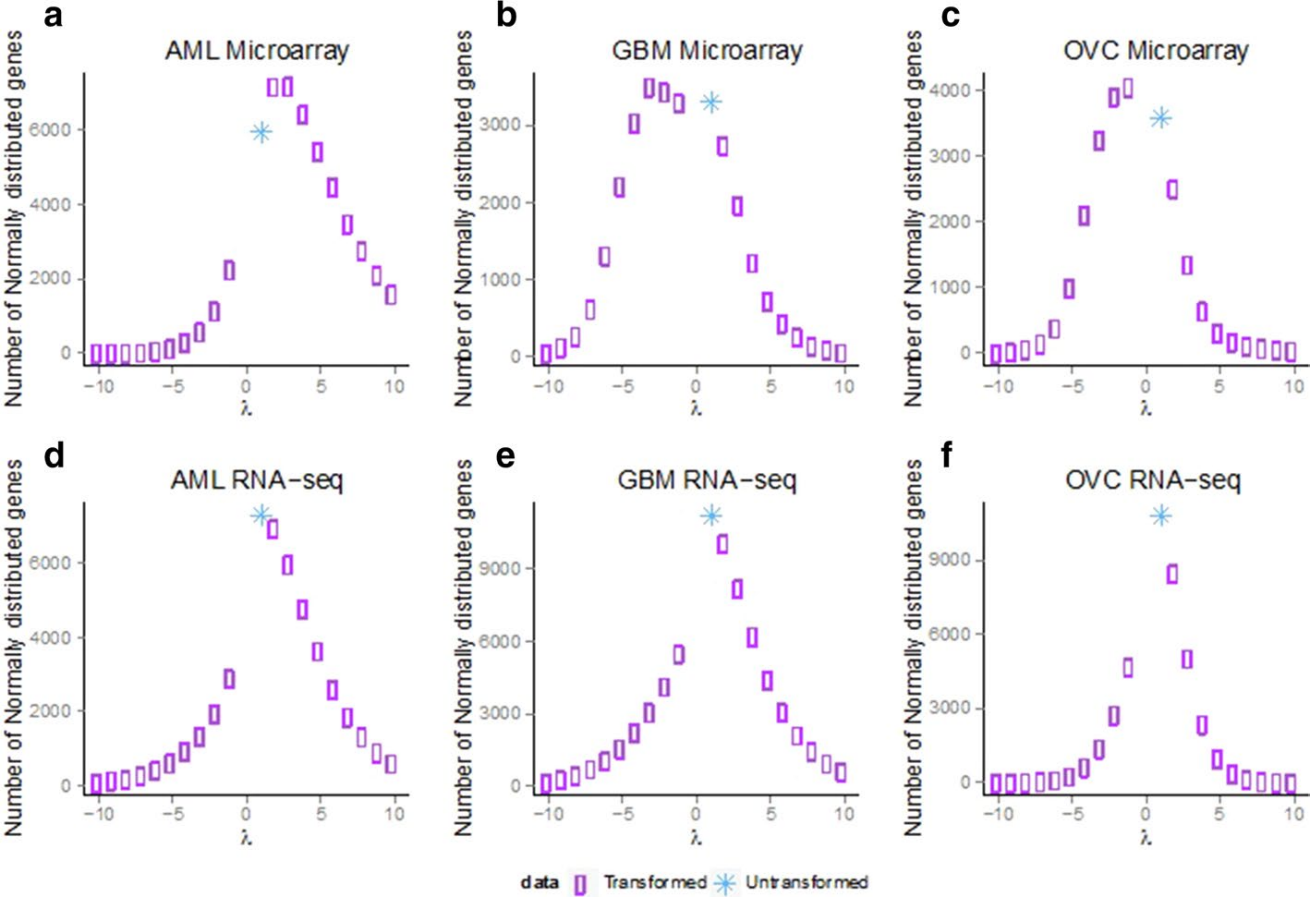
Box-Cox transformation applied to Microarray and RNA-seq datasets. The Box-Cox transformation $\frac{{x^{\lambda } - 1}}{\lambda }$ with $\lambda = - 10, \cdots ,10$ was applied to the three microarray and RNa-seq datasets to see if the number of Normally distributed genes was changing. The blue star corresponds to untransformed dataset and the purple rectangle to the transformed ones

Our results demonstrated an overwhelming proportion of non-Normal distributions, with the range of non-Normally expressed genes being 56.82 to 69.71% in the RNA-seq datasets for all three tumors. In applied statistics, a common procedure to induce Normality for seemingly non-Normal data is a Box-Cox transformation, and one could argue that applying these standard adjustments would restore Normality in the data. To investigate this, we applied the Box-Cox transformation with varying parameters $\lambda = - 10, \cdots ,10$ to both sets of gene expression data. For all three RNA-seq datasets, the maximum number of Normally-distributed genes was observed when the Box-Cox transformation was not applied. In other words, application of the Box-Cox transformation was not successful in converting the non-Normally-distributed genes into Normal ones across the parameter space that was used (Fig. 7). For the microarray datasets, the number of Normally-distributed genes did increase due to the Box-Cox transformation; however, the difference observed was small (Fig. 7a–c).

### Tumor purity does not influence variation in gene expression shape for the overall cancer transcriptome

To assess the influence of tumor purity, we used the information provided by TCGA that represented the pathologist’s estimate of purity for each patient sample and correlated this measure with gene expression. While it is possible to estimate tumor purity from gene expression data, in this case, we used the estimates that were calculated by the pathologist that were provided in the clinical data associated with each TCGA tumor sample. In general, minimal correlation was observed between the microarray, RNA-seq datasets and the tumor purity (Additional file 1: Figure S1). For the GBM RNA-seq dataset, no correlations between genes were statistically significant (adjusted *p* values < 0.001). For the remaining datasets, the number of significant correlations remained small relative to the total number of genes; where 377, 384 and 441 genes were statistically significant for GBM microarray, OV microarray and OV RNA-seq datasets respectively.

For the datasets where significant correlations were detected, we investigated these genes further to understand whether non-Normal distributions were likely to have an association with tumor purity. Specifically, for GBM microarray and OV microarray, the majority of the genes with a statistically significant correlation between tumor purity and gene expression were not categorized as Normal but were instead Gamma and bimodal respectively (see Additional file 1: Table S9A). For OV RNA-seq, the majority of these statistically significant genes were categorized as Normally-distributed in their gene expression profiles (see Additional file 1: Table S9A). We tested the association between Normally-distributed genes and their influence on tumor purity using Fisher’s exact tests. For the GBM microarray dataset, this association was not significant (*p* value > 0.05, see Additional file 1: Table S9B), but for both OV microarray and OV RNA-seq datasets, a statistical association was observed (*p* value = 0.0197, 0.00101, respectively). Interestingly, for the OV microarray dataset, this result points to non-Normally distributed genes having higher odds of also being influenced by tumor purity (i.e. they were also genes that were statistically significant between tumor purity and gene expression) (see Additional file 1: Table S9C). In contrast, for the OV RNA-seq dataset, this result showed the opposite—that Normally-distributed genes had a higher odds of also being influenced by tumor purity (see Additional file 1: Table S9D). Whether these results reflect different regulatory relationships that are tumor or platform-specific requires further investigation. It is important to recognize though that the gene numbers under analysis are relatively small, e.g. for the OV microarray dataset, 49 genes are Normally distributed and significant for tumor purity, and for the OV RNA-seq dataset, 226 genes are in this category. Therefore, it is challenging to draw trends from these small numbers about influence of tumor purity and distribution shape.

### Most distributions demonstrate significant enrichment for unique biological processes

To investigate any underlying trends in regulation, we also inspected whether genes belonging to a specific distribution category were enriched for unique biological processes. For each patient cohort dataset, over-representation analysis using Gene Ontology (GO) biological processes (BP) was applied to the list of genes assigned to each distribution (see Methods). There was a wide variety in the numbers of significant terms that were returned for each distribution and tumor type (see Additional file 1: Table S10). This was unsurprising, given that the number of genes assigned to each distribution was also quite variable (see Additional file 1: Table S1). Interestingly, we saw that some distribution categories had GO:BP terms that were unique and not significantly represented in other categories for a given tumor type (see Additional file 1: Table S10). Given the wide spread in the number of unique significant GO:BP terms, ranging from zero to 282, we chose to visualize these terms by reporting the top five most significant GO:BP terms for each distribution category in each patient cohort dataset (see Additional file 1: Table S11). For some distribution categories, no significant GO:BP terms that were unique were reported. However, each datasets had at least one distribution category that had significant GO:BP terms that were uniquely represented. Of note, we did not observe any clear overlap in the GO:BP terms for the same tumor type across different technology platforms e.g. the AML microarray results (Additional file 1: Table S11A) versus the AML RNA-seq results (Additional file 1: Table S11D). This likely reflects the that fact that different sets of genes were quantified and assigned to these distribution categories for the microarray and RNA-seq datasets.

## Discussion

Our study illustrates just how diverse distributions can be in cancer transcriptomes. We often take for granted that genes follow the same expression distribution and that it is their population-level summary statistics, like the average gene expression, that will identify key regulators of a phenotype or disease. While these summary statistics are indeed important, we showed that modeling other features of the expression distribution can also provide regulatory information. Fundamentally, the results of our study are significant because they force us to confront the fact that a gene’s expression profile cannot simply be summarized by a single statistical distribution. We showed how incorporating the shape of the expression distribution provided a means to identify genes with prognostic value for patient survival status that were not detected using conventional approaches that assume all genes are symmetrically distributed in a patient cohort. Moreover, we showed that using this shape information of the expression distribution resulted in a more accurate classification of patient survival time.

Throughout the history of science, the Normal distribution has been a ubiquitous feature in many forms of data analysis. Part of this ubiquity can be attributed to the central limit theorem (CLT), which explains how the average value of a variable will approximately follow a Normal distribution, regardless of the underlying data distribution. Validity of the CLT is dependent upon the design of the data being sufficiently large and the data points having been sampled independently from the same population. Because of the CLT, standard statistical methods typically have some degree of inbuilt robustness so that they are generally able to produce valid inferences even in the presence of some non-Normal data but this does not apply to all situations. In reality, deviations from Normality do exist in the data, but the extent of these deviations is not commonly assessed. For the entire transcriptome, this means that genes with expression profiles that more closely resemble a Normal distribution will be more easily detectable by standard statistical methods compared to genes that have a different expression distribution. This kind of bias means many genes may be being overlooked or down-weighted because we are not stopping to first evaluate the prevalence of different distributions [16].

Attention to non-Normality in gene expression has so far yielded some valuable insights in cancer biology. For instance, in a patient cohort, genes with distinct on and off transcriptional states followed a Bimodal distribution and have been detected in a variety of different tumor types [17]. These switch-like genes have been shown to identify patients subgroups with different rates of survival [18] or distinguish between extremely aggressive forms of tumors [19]. More recently, Piqué et al. [20] demonstrated how bimodal distributions in TCGA breast cancer data identified potential oncogenes for patient subgroups, including the gene *CBX2* which was shown to promote cancer cell growth in MCF-7 breast cancer cells. During the early development of statistical models for cDNA microarrays, Newton et al. [21] adopted a Gamma distribution to estimate significance of gene expression ratios. Despite the mathematical advantages of using the Gamma distribution, this study observed that the overall fit of the Gamma distribution to the entire transcriptome was relatively poor and the use of the distribution was discarded in favor of other more tractable distributions. If we interpret this finding from a different perspective, it is interesting to note that some genes that Newton et al. [21] surveyed had expression profiles that showed a good fit to a Gamma distribution, while others did not.

The search for other non-Normal distributions in the transcriptome remains limited despite the fact that these distributions have the potential to model rare regulatory events in large patient cohorts with more flexibility than a Normal distribution. Non-Normal distributions that are asymmetric and skewed can more accurately model genes spanning a range of aberrant expression for an extreme group of individuals than a symmetric distribution can [22]. Such long-tailed aberrations could reflect DNA sequence or copy number variation, different isoforms or alternative splicing patterns. Non-genetic factors at the environmental or epigenetic level may also drive the appearance of different sub-groupings of gene expression in the patient cohort.

The classification of genes into their respective expression distribution shapes may provide an avenue to integrate data of different genomic types such that regulatory mechanisms can be studied more fruitfully. For example, in the promoter region upstream of a gene with a bimodal gene expression distribution, a polymorphism may exist such that patients in one expression mode have this mutation, while patients in the other expression mode do not. Similarly, genes with asymmetric expression distributions may be a product of patients who share largely the same genomic features with a separate minority of patients whose outlier gene expression values reflect differences in methylation, alternative splicing or other regulatory events. Integration of clinical patient data with other genomic data types based on the shape of the distribution, whether it be for gene expression or DNA methylation, may be a more realistic way to identify significant relationships. This is because summary statistics are derived from the total population, i.e. an average expression assumes that all patients will have approximately, a specified level of gene expression.

A major focus of this study was to understand the prevalence of different distributions that were represented in a cancer transcriptome, of which the Normal distribution is one of the six distributions that was under consideration. While gene expression as measured by RNA-seq is usually represented by a negative binomial distribution, the broader question is whether the negative binomial distribution is relevant for all genes in the RNA-seq dataset as opposed to other possible distributions being prevalent amongst all genes too. To make our study as comprehensive as possible, we included data from at least two technology platforms. Since both RNA-seq and microarray datasets were profiled for the same set of TCGA patients, this was an elegant design to capitalize on for this study. Because gene expression as measured by microarrays is typically modelled as continuous data (and sometimes thought to be Normally-distributed), we nominated a diverse panel of six distributions that were applied to both RNA-sequencing and microarray datasets for consistency. We also only chose tumor types where a large number of patient samples were available. This larger sample size helps to improve the validity of approximating count data with continuous distributions. It is interesting to note that for the RNA-sequencing datasets, the percentage of genes with a Normal distribution increased for all three tumor types compared to the microarray datasets (Fig. 3c).

One aspect of this study was to investigate how assumptions based on the gene expression distribution shape impacted the performance of survival analysis models. It is worthwhile recognizing that the use of thresholds to evaluate survival analysis differences is inherently arbitrary. A previous study took a broader view into investigating how threshold-based cut-offs versus more robust, less arbitrary approaches such as the concordance index (C-index), D-index, and K-means performed for survival analysis models based on gene expression data sets from TCGA [23]. Raman et al. also contrasted performance of these metrics against the distribution-based ones that were included in this study. Results from these comparisons showed that the C-index, D-index, and k-means had the strongest performance overall. The splits determined by the distribution-based assumptions were not amongst the best performers. This result suggests that for the most robust performance, survival analysis models should use metrics that identify the most appropriate data-specific split like the C-index, rather than rely on population-specific, pre-determined thresholds like quartiles.

The assumptions of gene expression distribution shape that were tested in this study are inherently tied to assumptions that are made about Normality. For example, the shape assumption permits distributions to be Normal (see Fig. 4a) or any of the non-Normal options (Bimodal, see Fig. 4c or asymmetric like the Gamma or Lognormal, see Fig. 4b) and adjusts the cut-offs required to build the statistical classifiers depending on the distribution shape of the gene. Implicitly built into the symmetric assumption is that only one type of distribution is permitted because only one set of cut-offs for the classifier are used (equal tails of the distribution based on an assumption of symmetry/Normality) regardless of whether the gene is Normally-distributed or not. Each non-Normal distribution contributes a specific role towards determining the appropriate cut-offs (e.g. Bimodal reflects the split between the two modes, asymmetric distributions like Gamma and Lognormal reflects an one-tailed cut-off split). Hence, the tests performed under the shape assumption demonstrated how incorporating non-Normal distributions improved the statistical classification process (Fig. 6) over the symmetric one.

Genome sequencing projects like TCGA, but also ICGC, HAPMAP, ENCODE, and 1000 Genomes have given us a deeper appreciation for how heterogeneous human populations are with respect to genomic features. In light of this, it seems overly simplistic to assume that all key regulators will be found by correlating different data types on the assumption that all patients in the population will exhibit similar levels of the variable of interest. Instead, a more comprehensive approach may be based on identifying subgroupings of patients that share similar levels of a variable, and investigating whether there are correlations with other genomic features. Similarly, investigating how these gene expression distributions manifest at the single cell level within a tumor sample may also be instructive for understanding the contributions of different genes toward cancer growth and maintenance.

## Conclusions

This study identified the prevalence of genes with non-Normal gene expression distributions within cancer patient cohorts for AML, OV, and GBM from TCGA. Regardless of the technology platform, at least 50% of the cancer transcriptome was classified into one of five non-Normal distributions, including Cauchy, bimodal, Gamma, and Lognormal distributions. We tested the utility of incorporating assumptions based on the gene expression distribution into survival analysis models for the three cancer patient cohorts. Our results indicate that prognostic genes identified based on consideration of the shape of the distribution were different from those identified through more standard assumptions. These shape-based prognostic markers provided functional insights into cancer biology that were not detected using genes identified from standard approaches. Moreover, classifying patients based on poor versus good survival based on assumptions of gene expression shape resulted in higher performance than standard assumptions. This study has shown how subgroupings can be identified by considering the shape of the expression distribution and highlighted the value that can stem from this, both in terms of functional interpretations and the improved performance in statistical classification. More generally, the approaches used in this study provides a natural way to model heterogeneity under an explicit statistical framework. The results from this study raise new questions about the role of shape-based modeling for gene expression data.

## Methods

### The Cancer Genome Atlas datasets

Data were sourced from The Cancer Genome Atlas (TCGA, http://cancergenome.nih.gov/). The acute myeloid leukemia (AML) dataset had 197 samples with microarray data, and 173 samples with RNA-seq data. The glioblastoma multiforme (GBM) had 549 samples with expression data, 169 samples with RNA-seq data. The ovarian serous cystadenocarcinoma (OV) was used only for expression and RNA-seq data, and had 586 samples, respectively. For gene expression, the level 2 data on U133A (with 22,277 probes corresponding to 12,496 genes) for glioblastoma, ovarian and lung, and U133_plus_2 (with 54,613 probes corresponding to 19,850 genes) for AML were used in this study. For RNA-seq, for AML, GBM and OV, we used the level 3 IlluminaHiSeq_RNAseqV2 with a total of 20,531 genes. The expression levels from the RNA-seq datasets were reported as RSEM [24] raw counts that were quantile normalized for each cohort. We downloaded the clinical data corresponding to the microarray and RNA-seq datasets. From these files, the survival time (see summary in Additional file 1: Table S2) and the tumor purity estimated by a pathologist (for GBM and OV) were used. The missing values in survival time were omitted from all statistical analyses.

### Data preprocessing

For all microarray and RNA-seq data, gene expression values were log_2_-transformed. For RNA-seq, we filtered the genes by removing those with more than 25% of the samples with values less than 1 on the log_2_-transformed scale. GBM and OV datasets were batch-corrected using the function ComBat from the R package sva (version 3.14.0). We used the annotation R package hgu133a.db (version 3.1.3) for the GBM and OV datasets and hgu133plus2.db (version 3.1.3) for AML. To resolve multiple probes mapping to a unique gene identifier, we calculated the average of all expression values from probes mapping to the same gene symbol.

### Classifying genes into different distributions

For testing Normality and Lognormality, we used the Shapiro test from the R package stats (version 3.2.2) [25] with a threshold of 0.01 on the data and log of the data respectively. For Pareto, Gamma and Cauchy, the Kolmogorov–Smirnov test [26] was applied.

For this test, we needed to set parameter values. For Pareto and Gamma, the parameters were estimated with the Maximum Likelihood Estimates (MLE). For the MLE of Gamma, we used the rGammaGamma R package (version 1.0.12.). For Cauchy, the two parameters were set as the median and the interquartile range.

As we are estimating the parameters directly on the dataset, we applied a parametric bootstrap to estimate the final *p* value. This idea of resampling to find the null distribution of the test statistics when estimating the parameters is based on the Lilliefors test [27]. The threshold for the final *p* value was set to 0.01 for the significance. For testing Bimodality, we computed the Bimodality Index [28] from the R package ClassDiscovery (version 3.0.0.) and kept every gene and locus having a score bigger than 1.1.

In order to have a faster algorithm, we first test if a gene is bimodal, if yes it is classified as so and removed from the list and otherwise the other distributions are tested and the best was chosen (see Fig. 2). As the Lognormal, Pareto and Gamma distribution are defined on positive values, they were tested only on genes having all their values greater than zero. If we have an equality between two distributions the order of classification is as followed: (1) Normal, (2) Lognormal, (3) Cauchy. Genes that fall in neither category are classified as unknown and the distributions sets are disjoint meaning a gene cannot be assigned to more than one distribution.

### Evaluating differences in survival time

In order to test the difference between two survival curves, we used the log-rank test from the R package survival (version 2.38.3) with a threshold of 0.05. To estimate the survival curves, the Kaplan–Meier estimate was used.

### Over-representation analysis

The Bioconductor package GOstats (version 2.48.0) was used to test for the over-representation of Gene Ontology Biological Processes that also included annotations from the Bioconductor package org.Hs.eg.db (version 3.7.0). *p* values were corrected for multiple testing using the p.adjust function with the Benjamini–Hochberg method. Statistical significance was set at 0.0001.

## Supplementary information

**Additional file 1.** Supplementary figures and tables.

## Data Availability

All data used to support the conclusions in this study is publicly available from The Cancer Genome Atlas (TCGA, http://cancergenome.nih.gov/).

## Abbreviations

AML: Acute myeloid leukemia
BP: Biological process
CLT: Central limit theorem
GO: Gene ontology
GBM: Glioblastoma multiforme
OV: Ovarian serous cystadenocarcinoma
RNA-seq: RNA-sequencing
TCGA: The Cancer Genome Atlas
