# Supplementary material for: The shape of gene expression distributions matter: how incorporating distribution shape improves the interpretation of cancer transcriptomic data
Source: BMC Bioinformatics. 2020 Dec 28;21(Suppl 21):562. doi: 10.1186/s12859-020-03892-w (PMC7768656; doi:10.1186/s12859-020-03892-w)
Supplement: Supplementary file 1 — Additional file 1. Supplementary figures and tables. [file 12859_2020_3892_MOESM1_ESM.docx]

**Supplementary Info**

**Figure S1. Correlation between tumor purity and gene expression datasets.** We computed the Spearman correlation between the pathologist estimates of tumor purity and the expression or beta values.These histograms represent the correlation coefficients with, in pink, the coefficients that were significant (p-values<0.01). **a** GBM Microarray with 377 significant coefficients, **b** OVC Microarray with 384 significant coefficients, **c** GBM RNA-seq with no significant coefficients, **d** OVC RNA-seq with 441 significant coefficients.


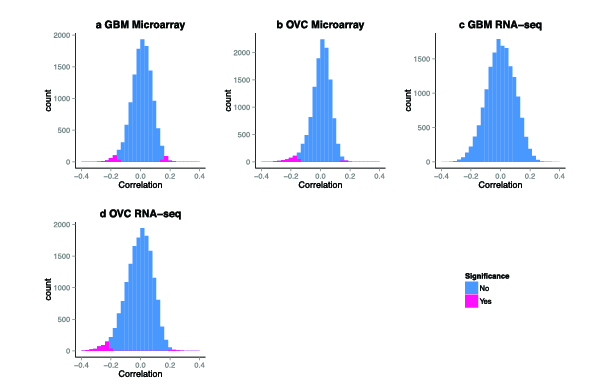


**Table S1. Number of genes and loci in each distribution for AML, GBM and OVC.** For the microarray dataset, AML was done on a hgu133plus2 platform which contains more probes than the hgu133plus2A platform that was used for GBM and OVC. To have a better comparison, we decided to report also the number corresponding to the intersection with the plus2A platform for AML (cf. AML (plus2A)).

|  | **Bimodal** | **Normal** | **Lognormal** | **Gamma** | **Cauchy** | **Pareto** | **Unknown** | **Total** |
| --- | --- | --- | --- | --- | --- | --- | --- | --- |
| **Microarray** |  |  |  |  |  |  |  |  |
| AML | 4,323  (21.20%) | 2,799  (13.73%) | 679  (3.33%) | 5,779  (28.34%) | 958  (4.70%) | 0  (0%) | 5,851  (28.70%) | 20,389 |
| AML (plus2A) | 2,113  (16.99%) | 1,809  (14.55%) | 451  (3.63%) | 3,925  (31.57%) | 637  (5.12%) | 0  (0%) | 3,499  (28.14%) | 12,434 |
| GBM | 847  (6.81%) | 1,681  (13.52%) | 1,319  (10.61%) | 2,714  (21.83%) | 1  (0.01%) | 0  (0%) | 5,872  (47.23%) | 12,434 |
| OVC | 1,065  (8.57%) | 1,881  (15.13%) | 1,653  (13.29%) | 2,973  (23.91%) | 0  (0%) | 0  (0%) | 4,862  (39.10%) | 12,434 |
| **RNA-seq** |  |  |  |  |  |  |  |  |
| AML | 1,637  (11.15%) | 4,447  (30.29%) | 683  (4.65%) | 3,591  (24.46%) | 899  (6.12%) | 0  (0%) | 3,424  (23.32%) | 14,681 |
| GBM | 1,747  (10.77%) | 6,779  (41.80%) | 1,391  (8.58%) | 3,975  (24.51%) | 341  (2.10%) | 0  (0%) | 1,983  (12.23%) | 16,216 |
| OVC | 1,081  (6.68%) | 6,990  (43.18%) | 1,717  (10.61%) | 3,841  (23.73%) | 4  (0.02%) | 0  (0%) | 2,554  (15.78%) | 16,187 |

**Table S2. Number of genes significant in survival time with expression data for a. Microarray and b. RNA-seq datasets.** The total number corresponds to the number of genes in each distribution for each expression data sets. The significance in survival time was assessed using the Log-rank test and a P-value < 0.05. The shape assumption classifies the five distributions into three classes: symmetric (Normal and Cauchy), Bimodal and asymmetric (Gamma and Lognormal). The symmetric assumption uses the symmetric method regardless of the specific distribution. The random assumption takes 20% of the samples in one group and compares them to the remaining 80%. In the second and third columns, the number in brackets corresponds to the number of genes that were found to be significant under both the shape and symmetric assumptions, and the shape and random assumptions, respectively.

**a** Microarray

|  | **Significant in Survival Time** | | | **Total Number of Genes** |
| --- | --- | --- | --- | --- |
|  | **With shape assumption** | **With symmetric assumption** | **With a random assumption** |  |
| **Normal** |  |  |  |  |
| AML | 2 | 0 | 0 | 2,799 |
| GBM | 6 | 1 | 0 | 1,681 |
| OVC | 0 | 0 | 0 | 1,881 |
| **Cauchy** |  |  |  |  |
| AML | 0 | 0 | 0 | 958 |
| GBM | 0 | 0 | 0 | 1 |
| OVC | 0 | 0 | 0 | 0 |
| **Lognormal** |  |  |  |  |
| AML | 0 | 0 | 0 | 679 |
| GBM | 14 | 0 | 0 | 1,319 |
| OVC | 2 | 0 | 0 | 1,653 |
| **Bimodal** |  |  |  |  |
| AML | 7 | 0 | 0 | 4,323 |
| GBM | 30 | 1 | 0 | 847 |
| OVC | 0 | 0 | 0 | 1,065 |
| **Gamma** |  |  |  |  |
| AML | 12 | 3 | 0 | 5,779 |
| GBM | 27 | 2 | 0 | 2,714 |
| OVC | 0 | 0 | 0 | 2,973 |
| **Number of Significant Genes** |  |  |  |  |
| AML | 21 | 3 | 0 |  |
| GBM | 77 | 4 | 0 |  |
| OVC | 2 | 0 | 0 |  |

**b** RNA-seq

|  | **Significant in Survival Time** | | | **Total Number of Genes** |
| --- | --- | --- | --- | --- |
|  | **With shape assumption** | **With symmetric assumption** | **With a random assumption** |  |
| **Normal** |  |  |  |  |
| AML | 9 | 5 | 0 | 4,447 |
| GBM | 0 | 0 | 0 | 6,779 |
| OVC | 3 | 3 | 0 | 6,990 |
| **Cauchy** |  |  |  |  |
| AML | 0 | 0 | 0 | 899 |
| GBM | 0 | 0 | 0 | 341 |
| OVC | 0 | 0 | 0 | 4 |
| **Lognormal** |  |  |  |  |
| AML | 4 | 0 | 0 | 683 |
| GBM | 0 | 0 | 0 | 1,391 |
| OVC | 0 | 1 | 0 | 1,717 |
| **Bimodal** |  |  |  |  |
| AML | 8 | 1 | 0 | 1,637 |
| GBM | 1 | 0 | 0 | 1,747 |
| OVC | 0 | 0 | 0 | 1,081 |
| **Gamma** |  |  |  |  |
| AML | 20 | 0 | 0 | 3,591 |
| GBM | 1 | 20 | 0 | 2,714 |
| OVC | 0 | 0 | 0 | 3,841 |
| **Number of Significant Genes** |  |  |  |  |
| AML | 41 | 6 | 0 |  |
| GBM | 2 | 0 | 0 |  |
| OVC | 3 | 4 | 0 |  |

**Table S3. List of genes significant in survival time using the shape assumption per distribution in a. AML Microarray, b. GBM Microarray, c. OVC Microarray, d. AML RNA-seq, e. GBM RNA-seq and f. OVC RNA-seq.**

**a. AML Microarray**

| **Distribution** | **Number of genes** |  | **List of genes** |
| --- | --- | --- | --- |
| **Normal** | 2 |  | GPRC6A, IGFBP7 |
| **Bimodal** | 7 |  | ADAM22, DEGS1, FAM155B, IMPACT, MOB2  MSMB, VWC2 |
| **Gamma** | 12 |  | ABCB11, ASAP1, CRYGA, CSHL1, DCN, EPOR,  GPCPD1, INPP5A, LAT, SLC38A5, SPDYE2, TRIM35 |

**b. GBM Microarray**

| **Distribution** | **Number of genes** |  | **List of genes** |
| --- | --- | --- | --- |
| **Normal** | 6 |  | KLF9, MMP15, MYLPF, PSMF1, SLC31A2, STX12 |
| **Lognormal** | 14 |  | APEX2, BRD7, COX4I1, CTCF, EIF3F, ETFB, GATA2  HAUS3, IRF2BP1, NOTCH2, PGLYRP4  RALYL, TEAD3, XPNPEP3, |
| **Bimodal** | 30 |  | ALOX12P2, APBA3, BDKRB1, CACNG2, CBFA2T2  CLDN5, CLK4, CRCP, DFNA5, EPB41L1, GDF2  GNRHR, GTF3A, IFNAR2, KCNMB1, KLHDC4  LOC79160, MAP1LC3C, METTL1, PPCS, RALBP1  RARG, RBFOX1, RND1, SLC6A15, TAB1, TREML2  TRIM36, UBAP2L, XRCC3 |
| **Gamma** | 27 |  | AHSG, ALDOB, ARHGEF40, CAPN15, CENPJ, EIF3M  GADD45A, LRP5, MAP3K12, MED22, MEF2C, MPZL1  NCR3, PECR, PPP2R5A, PRLR, RANBP10, RPL24  SAP30, SEC11A, SEPP1, SEPT07, SMNDC1, SYK  TRIM28, TRPC6, TSPYL5 |

**c. OVC Microarray**

| **Distribution** | **Number of genes** |  | **List of genes** |
| --- | --- | --- | --- |
| **Lognormal** | 2 |  | HNRNPF, ZFHX2 |

**d. AML RNA-seq**

| **Distribution** | **Number of genes** |  | **List of genes** |
| --- | --- | --- | --- |
| **Normal** | 9 |  | CDCA2, ECE1, KIAA1462, LTB, RGL1, RNASE1  SKA1, SLC1A3, SYNPO2L |
| **Lognormal** | 4 |  | DUSP7, MTCH1, PPAPDC1B, SLC1A5 |
| **Bimodal** | 8 |  | CD52, CPNE8, DOCK1, KIAA1958, LOC100130264  RHOBTB3, SPATS2L, SYTL4 |
| **Gamma** | 20 |  | ADSS, CAP1, CLINT1, CUX1, DKFZP586I1420  GALNT7, GUSBP3, HMMR, ITFG1, LARP1  NAP1L1, PNPLA6, RUSC1, SCAMP1, SMAD2  SUDS3, TLK2, TUBGCP3, UBE2C, UBQLN1 |

**e. GBM RNA-seq**

| **Distribution** | **Number of genes** |  | **List of genes** |
| --- | --- | --- | --- |
| **Bimodal** | 1 |  | RPL39L |
| **Gamma** | 1 |  | PODNL1 |

**f. OVC RNA-seq**

| **Distribution** | **Number of genes** |  | **List of genes** |
| --- | --- | --- | --- |
| **Normal** | 3 |  | C11orf51, CXCL12, PPP1R13L |

**Table S4.** **Summary of the survival time for a. Microarray and b. RNA-seq.** The survival time is defined as the days to death for the samples that are dead and days to last follow up for the ones still alive. The number of NA relates to the number of samples when the days to death and the last follow up is unknown.

**a Microarray**

|  | **AML** | **GBM** | **OV** |
| --- | --- | --- | --- |
| **Vital status** |  |  |  |
| Dead | 131 | 462 | 306 |
| Alive | 66 | 86 | 280 |
| **Median time in days** | 365 | 361 | 896 |
| **Number of NA** | 11 | 4 | 6 |

**b RNA-seq**

|  | **AML** | **GBM** | **OV** |
| --- | --- | --- | --- |
| **Vital status** |  |  |  |
| Dead | 114 | 131 | 173 |
| Alive | 59 | 37 | 136 |
| **Median time in days** | 335 | 334 | 883 |
| **Number of NA** | 10 | 2 | 2 |

**Table S5. Functional enrichment analyses for genes whose expression distributions are associated with significant patient survival time for the TCGA AML cohort.** Using the Investigate Gene Sets tool from GSEA/MSigDB, we detected significantly enriched terms and pathways for the top 100 most significant in survival time genes using the shape assumption (**a.** Microarray and **c.** RNA-seq), and top 100 most significant in survival time genes using a regular symmetric assumption (**b.** Microarray and **d.** RNA-seq). We used a threshold of FDR q-value < 0.05 or top 10 (whichever is less) was applied, and we computed overlaps with the following sets, H: Hallmark gene sets, C2 : KEGG and REACTOME, C5 : GO biological process, C6 : oncogenic signatures. Blue cells denote terms that were observed for both shape and symmetric assumptions, yellow cells denote terms that were unique to the shape or symmetric assumption (comparison between **a.** and **b.** and between **c.** and **d.**).

**a** Microarray Shape assumption


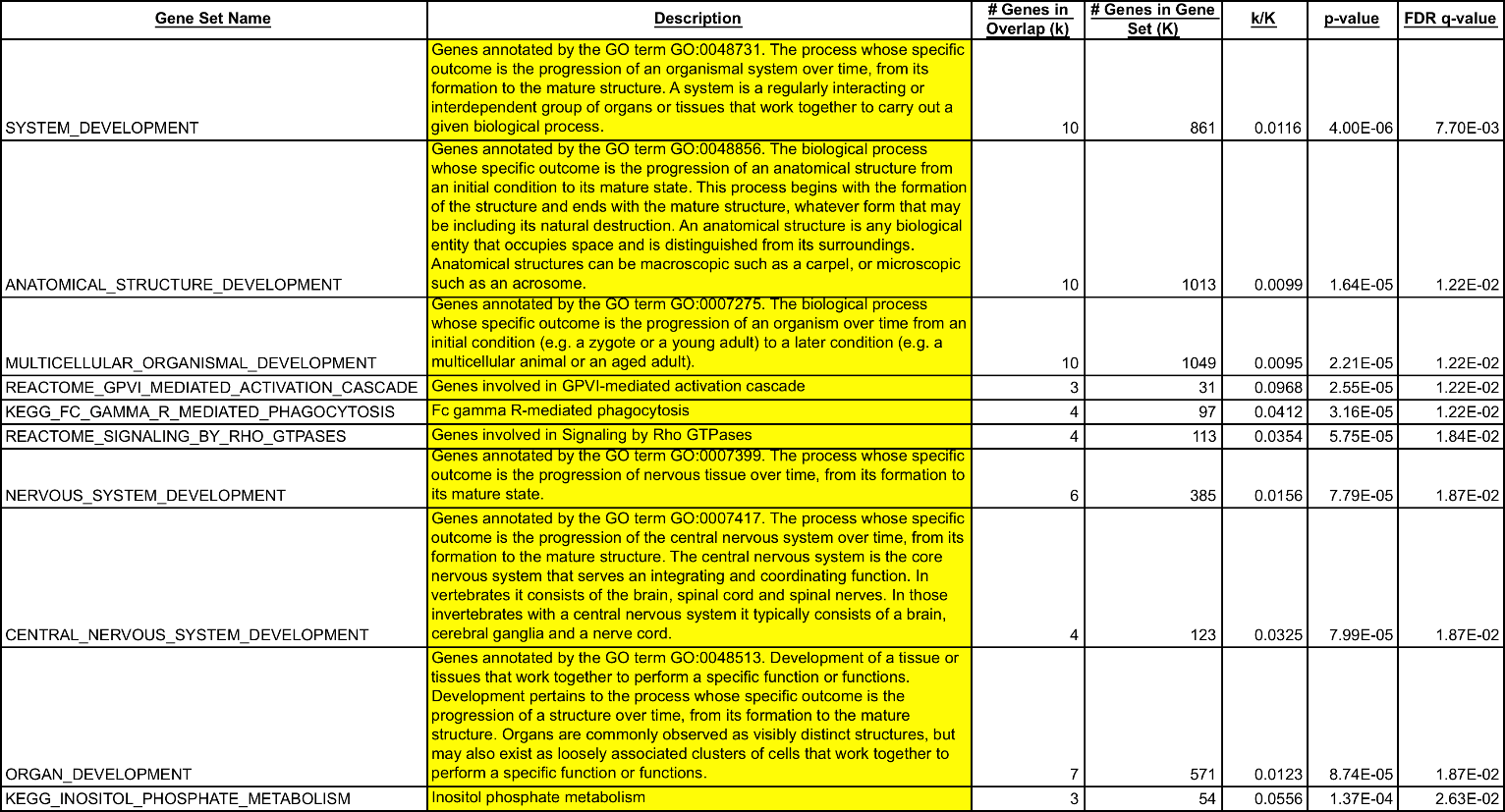


**b** Microarray Symmetric assumption


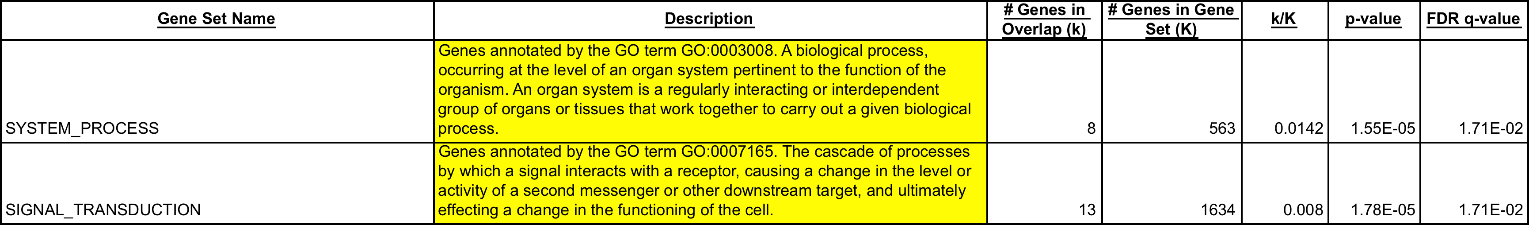


**Table S6. Functional enrichment analyses for genes whose expression distributions are associated with significant patient survival time for the TCGA GBM cohort.** Using the Investigate Gene Sets tool from GSEA/MSigDB, we detected significantly enriched terms and pathways for the top 100 most significant in survival time genes using the shape assumption (**a.** Microarray and **c.** RNA-seq), and top 100 most significant in survival time genes using a regular symmetric assumption (**b.** Microarray and **d.** RNA-seq). We used a threshold of FDR q-value < 0.05 or top 10 (whichever is less) was applied, and we computed overlaps with the following sets, H: Hallmark gene sets, C2 : KEGG and REACTOME, C5 : GO biological process, C6 : oncogenic signatures. Blue cells denote terms that were observed for both shape and symmetric assumptions, yellow cells denote terms that were unique to the shape or symmetric assumption (comparison between **a.** and **b.** and between **c.** and **d.**).

**a** Microarray Shape assumption


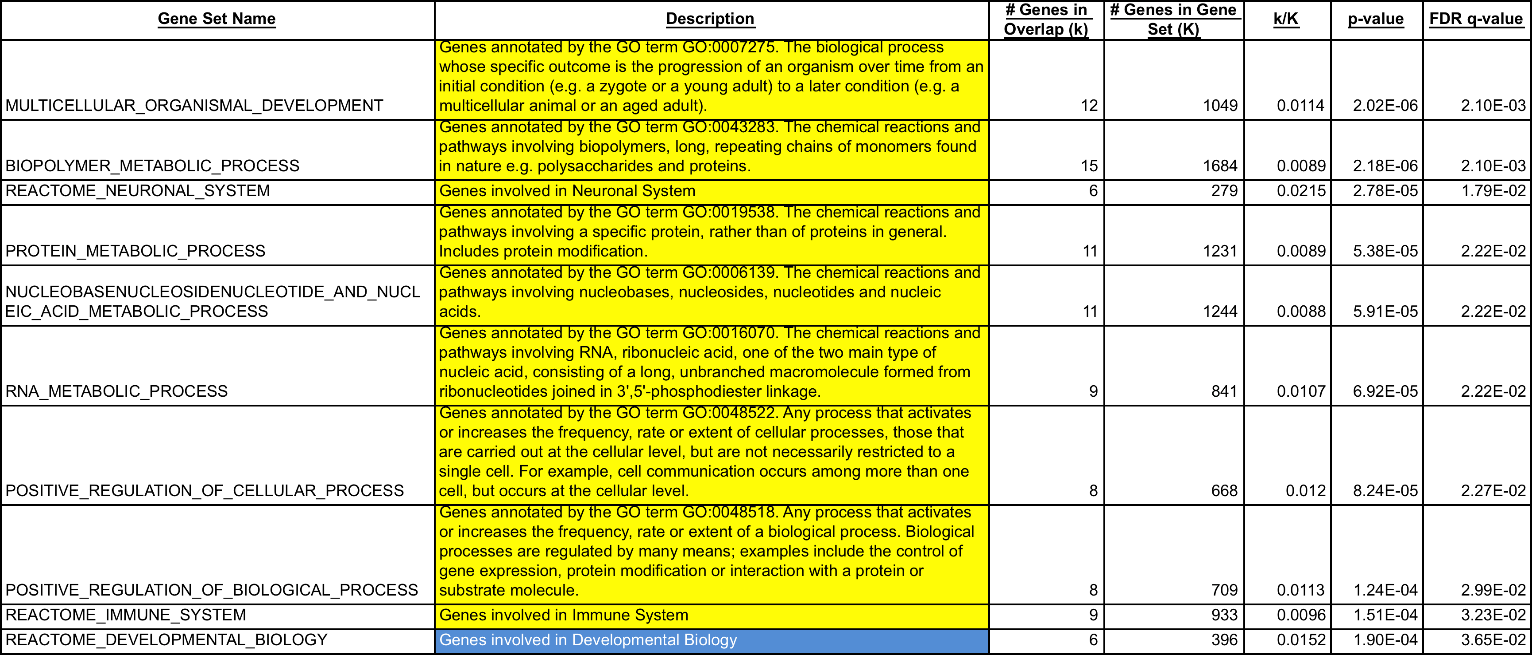


**b** Microarray Symmetric assumption


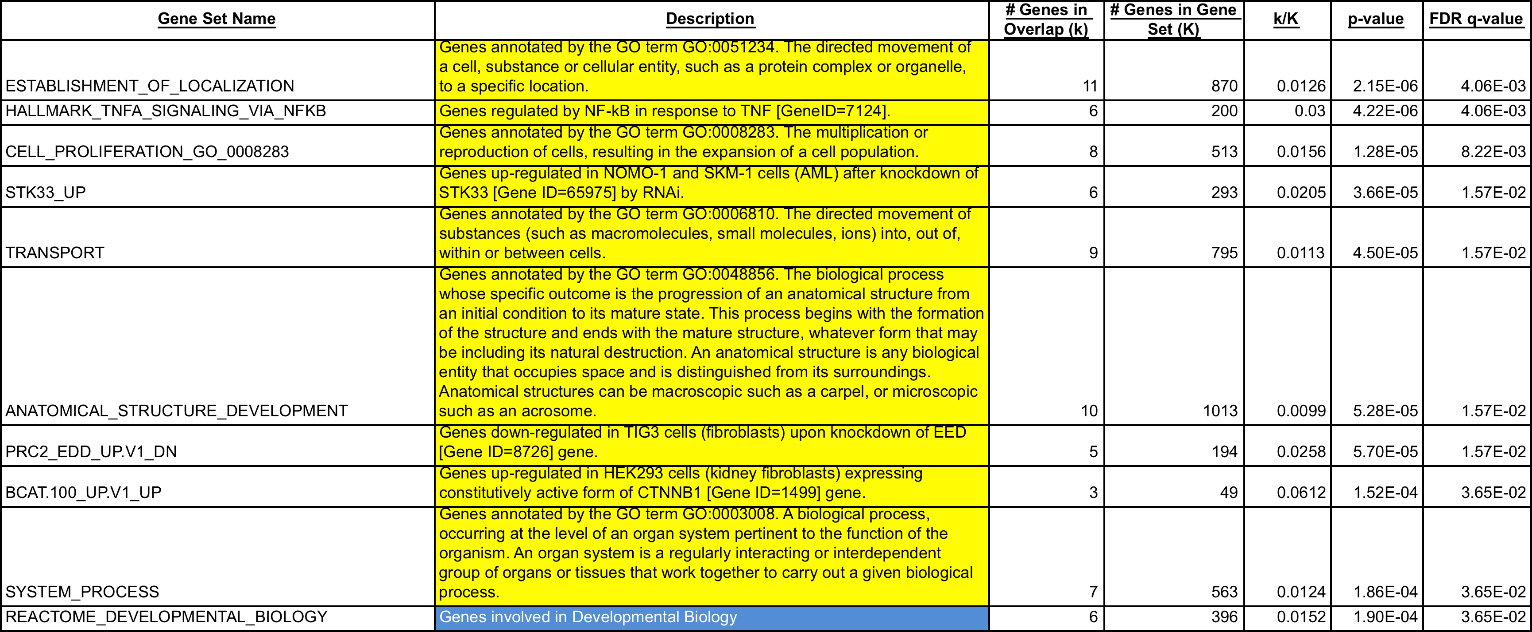


**c** RNA-seq Shape assumption


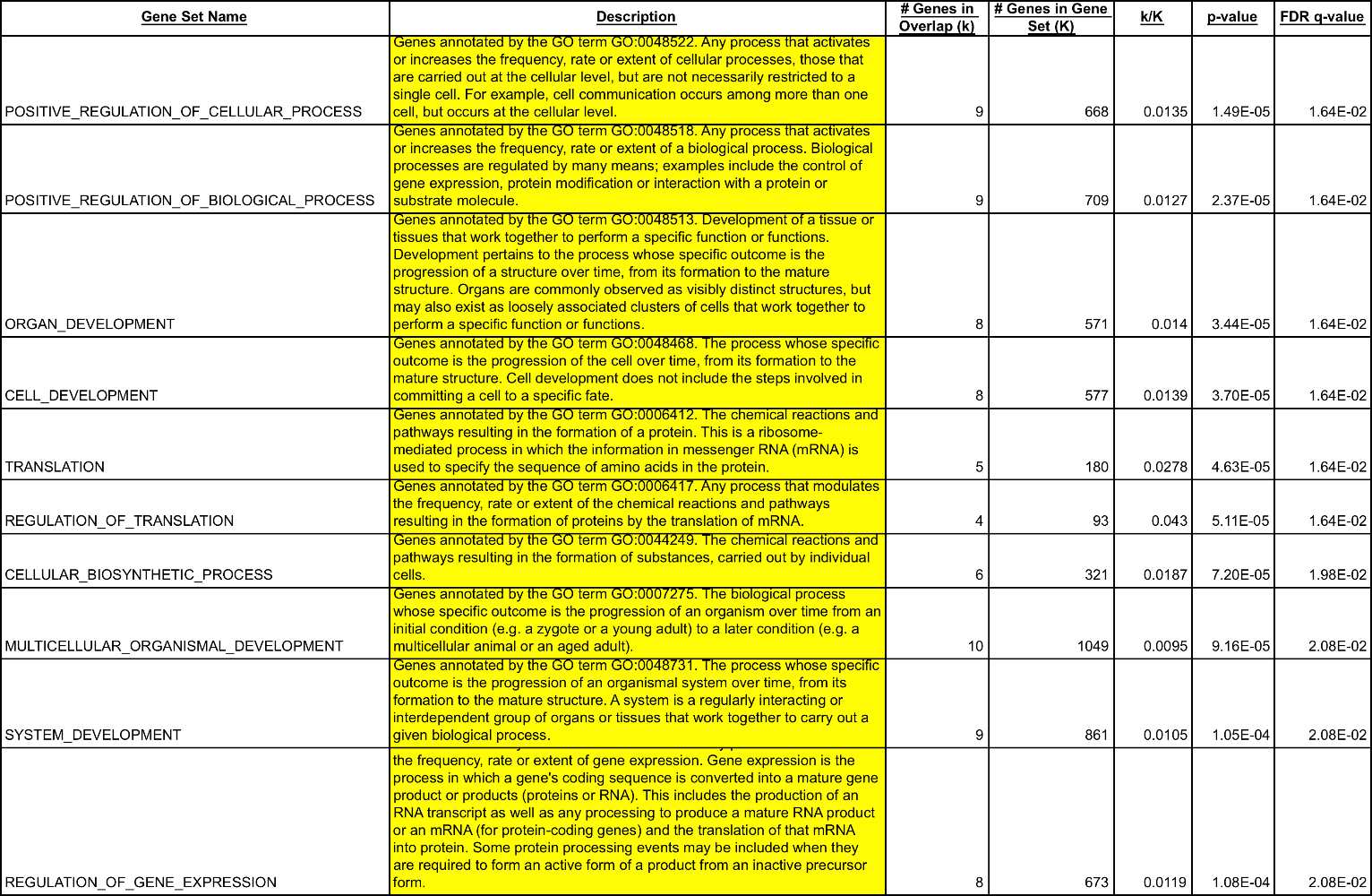


**d** RNA-seq Symmetric assumption


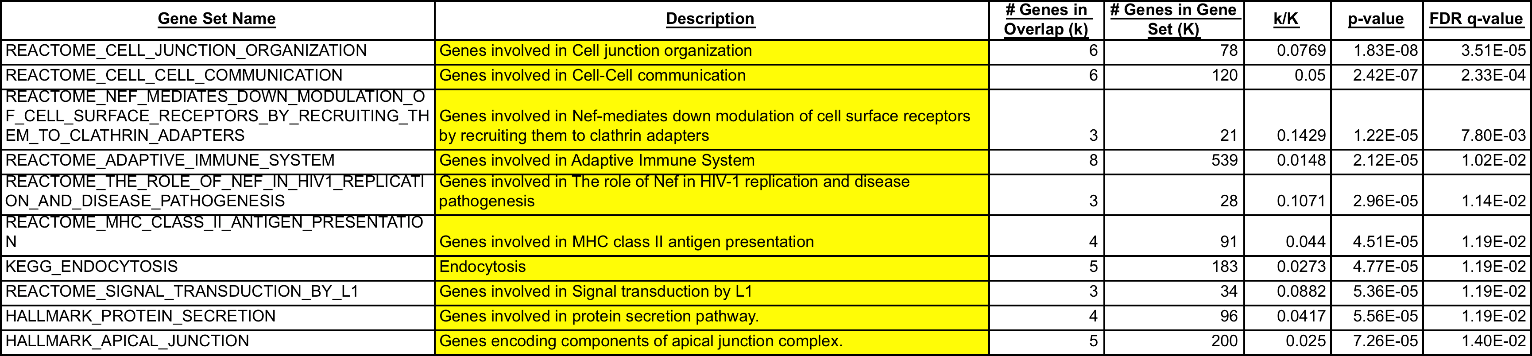


**Table S7. Functional enrichment analyses for genes whose expression distributions are associated with significant patient survival time for the TCGA OVC cohort.** Using the Investigate Gene Sets tool from GSEA/MSigDB, we detected significantly enriched terms and pathways for the top 100 most significant in survival time genes using the shape assumption (**a.** Microarray and **c.** RNA-seq), and top 100 most significant in survival time genes using a regular symmetric assumption (**b.** Microarray and **d.** RNA-seq). We used a threshold of FDR q-value < 0.05 or top 10 (whichever is less) was applied, and we computed overlaps with the following sets, H: Hallmark gene sets, C2 : KEGG and REACTOME, C5 : GO biological process, C6 : oncogenic signatures. Blue cells denote terms that were observed for both shape and symmetric assumptions, yellow cells denote terms that were unique to the shape or symmetric assumption (comparison between **a.** and **b.** and between **c.** and **d.**).

**a** Microarray Shape assumption


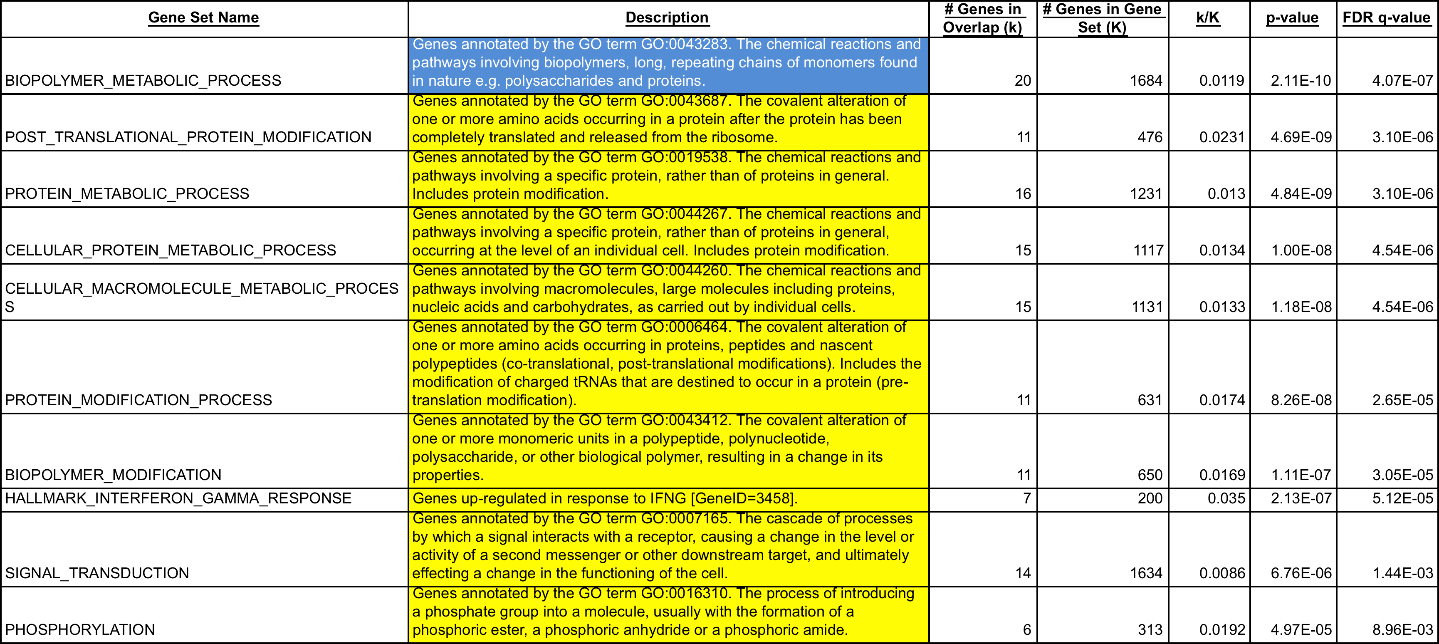


**b** Microarray Symmetric assumption


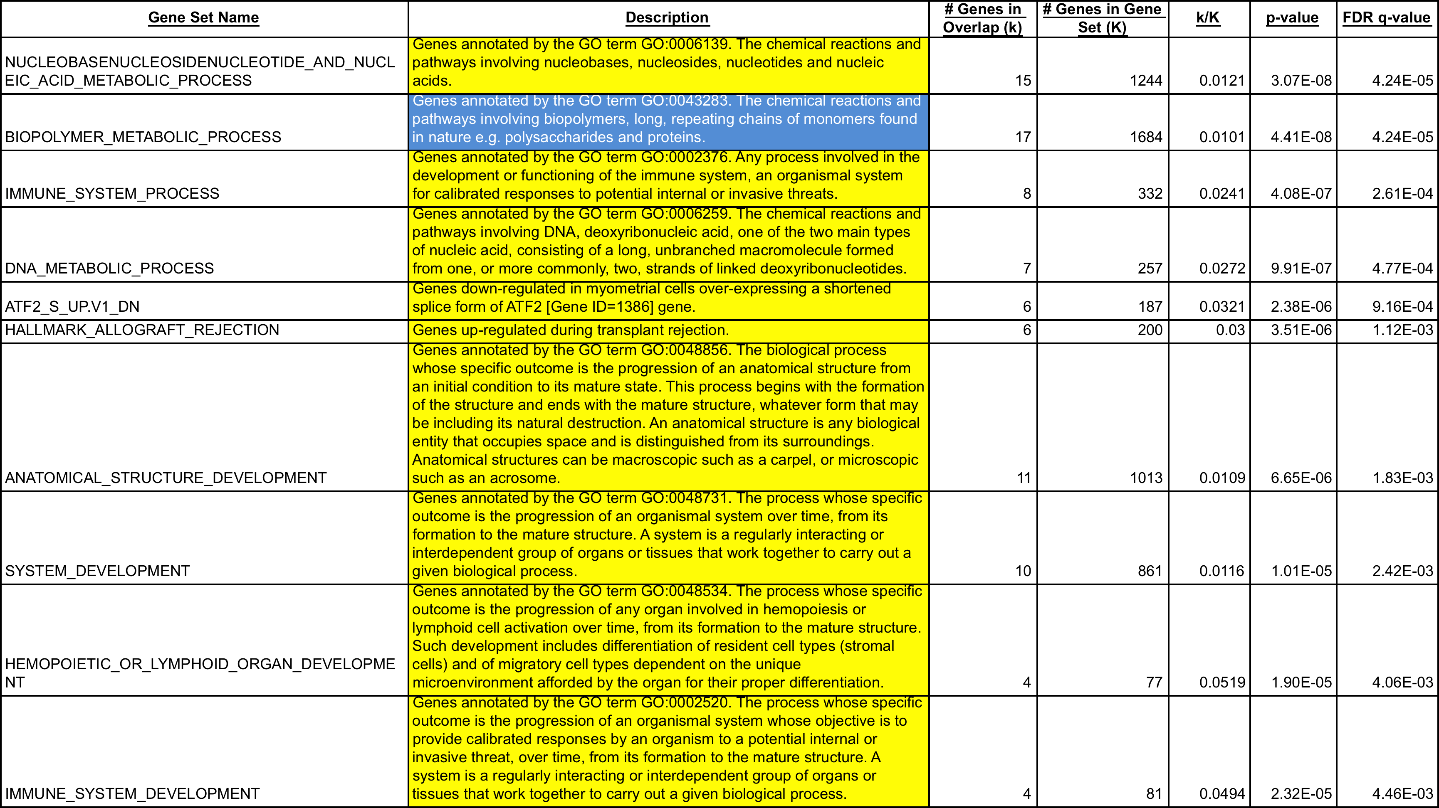


**c** RNA-seq Shape assumption


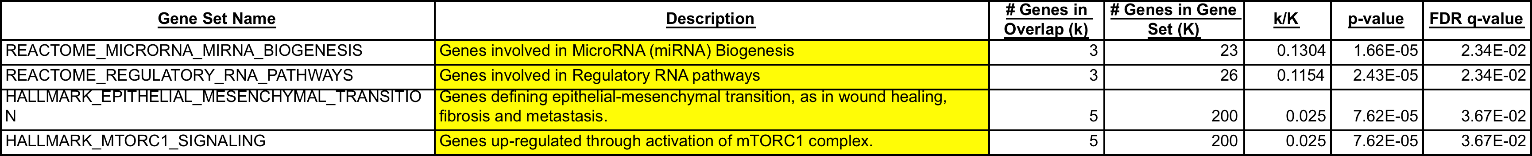


**d** RNA-seq Symmetric assumption


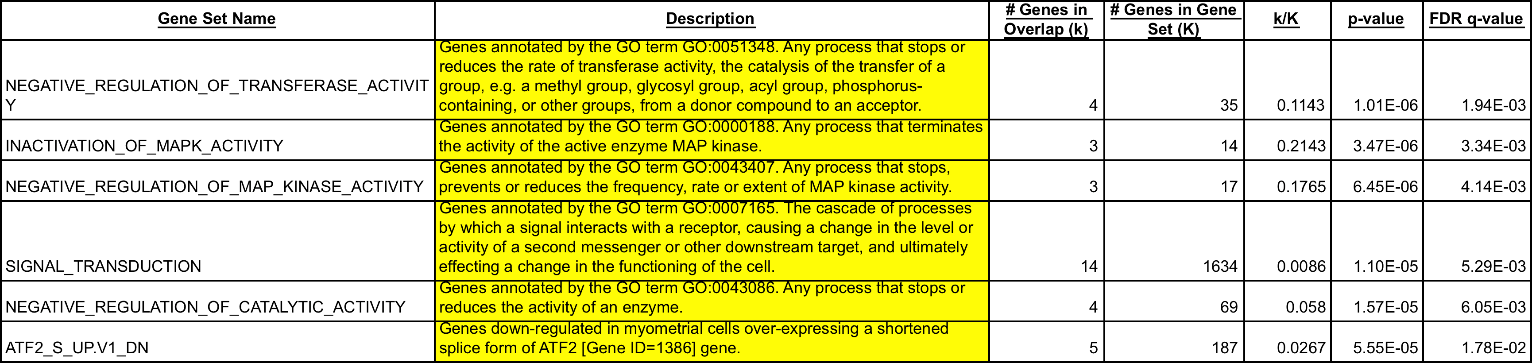


**Table S8.** **Overlap between genes that were previously identified in a prognostic signature and those appearing in our list of genes with different expression distributions for the TCGA AML RNA-seq data set.** No overlap was found with the TCGA AML microarray dataset.

| **Gene Expression Distributions** | **Prognostic Signatures** | | | | | | | |
| --- | --- | --- | --- | --- | --- | --- | --- | --- |
|  | Valk et al. (2004)^1^ | Bartholdy et al.  (2014)^2^ | Marcucci et al. (2011)^3^ | Gentles et al. (2010)^4^ | Eppert et al. (2011)^5^  Phenotypic Signature | Eppert et al. (2011)^5^  Leukemia Stem Cell Signature (LSC-R) | Eppert et al. (2011)^5^  Hematopoietic Stem Cell Signature (HSC-R) | Li et al. (2013)^6^ |
| **Normal** | - | RNASE1 | - | LTB | - | - | - | - |
| **Lognormal** | - | - | - | - | - | - | - | - |
| **Bimodal** | - | - | - | - | - | - | - | - |
| **Cauchy** | - | - | - | - | - | - | - | - |
| **Gamma** | - | TLK2 | - | - | - | - | - | - |
| **Total Overlap** | 0 | 2 | 0 | 1 | 0 | 0 | 0 | 0 |
| **Number of Genes in Signature** | 175 | 561 | 17 | 50 | 47 | 47 | 143 | 24 |

1. Valk et al. Prognostically Useful Gene Expression Profiles in Acute Myeloid Leukemia. N Engl J Med 2004; 350:1617-28.
2. Bartholdy et al. HSC commitment–associated epigenetic signature is prognostic in acute myeloid leukemia. 2014.
3. Marcucci et al. The prognostic and functional role of microRNAs in acute myeloid leukemia. Blood. 2011.
4. Gentles et al. Association of a Leukemic Stem Cell Gene Expression Signature With Clinical Outcomes in Acute Myeloid Leukemia. Journal of the American Medical Association. 2010.
5. Eppert et al. Stem cell gene expression programs influence clinical outcome in human leukemia. 2011.
6. Li et al. Identification of a 24-Gene Prognostic Signature That Improves the European Leukemia Net Risk Classification of Acute Myeloid Leukemia: An International Collaborative Study. Journal of Clinical Oncology. 2013.

**Table S9. Evaluating the Association Between Gene Expression Shape and Tumor Purity.**

**A. Distribution Counts for Genes that Had Statistically Significant Correlations Between Gene Expression and Tumor Purity.** The breakdown of genes that had significant influence of tumor purity on gene expression (adjusted P-value < 0.001) into their respective distribution shape categories.

|  | **Bimodal** | **Normal** | **Lognormal** | **Gamma** | **Cauchy** | **Pareto** | **Unknown** | **Total** |
| --- | --- | --- | --- | --- | --- | --- | --- | --- |
| GBM  Microarray | 60 | 71 | 39 | 83 | 0 | 0 | 124 | 377 |
| OV Microarray | 104 | 49 | 39 | 69 | 0 | 0 | 123 | 384 |
| OV  RNA-seq | 41 | 226 | 31 | 81 | 0 | 0 | 62 | 441 |

**B. Testing the Association Between Normally-Distributed Genes and Genes that were Significantly Influenced by Tumor Purity for the GBM Microarray Dataset.** Please note that these counts do not include Unknown genes. The Fisher’s exact test was applied with a two-sided alternative hypothesis.

| **GBM Microarray** | **Normal** | **Non-Normal (excluding Unknowns)** |
| --- | --- | --- |
| **Significant (Tumor Purity vs Gene Expression)** | 71 | 182 |
| **Not Significant** | 1610 | 4699 |
| **Odds Ratio Estimate** | 1.14 | |
| **P-value** | 0.378 | |

**C.** **Testing the Association Between Normally-Distributed Genes and Genes that were Significantly Influenced by Tumor Purity for the OV Microarray Dataset.** Please note that these counts do not include Unknown genes. The Fisher’s exact test was applied with a two-sided alternative hypothesis.

| **OV Microarray** | **Normal** | **Non-Normal (excluding Unknowns)** |
| --- | --- | --- |
| **Significant (Tumor Purity vs Gene Expression)** | 49 | 212 |
| **Not Significant** | 1832 | 5479 |
| **Odds Ratio Estimate** | 0.691 | |
| **P-value** | 0.0197 | |

**D.** **Testing the Association Between Normally-Distributed Genes and Genes that were Significantly Influenced by Tumor Purity for the OV RNA-seq Dataset.** Please note that these counts do not include Unknown genes. The Fisher’s exact test was applied with a two-sided alternative hypothesis.

| **OV RNA-seq** | **Normal** | **Non-Normal (excluding Unknowns)** |
| --- | --- | --- |
| **Significant (Tumor Purity vs Gene Expression)** | 226 | 153 |
| **Not Significant** | 6764 | 6490 |
| **Odds Ratio Estimate** | 1.42 | |
| **P-value** | 0.00101 | |

**Table S10. Significant GO:BP Terms from Over-representation Analysis on Genes Classified in Each Distribution.** The analysis was computed using GOstats with P-values adjusted using the Benjamini-Hochberg method and significance threshold set at adjusted P-value < 0.0001. NA indicates that there were not enough genes assigned to this distribution to run an over-representation analysis.

1. **AML microarray dataset**

|  | Bimodal | Normal | Lognormal | Gamma | Cauchy |
| --- | --- | --- | --- | --- | --- |
| *Number of Significant GO:BP Terms* | 0 | 13 | 0 | 154 | 0 |
| *Number of Significant GO:BP Terms Unique to Distribution* | 0 | 0 | 0 | 141 | 0 |

1. **GBM microarray dataset**

|  | Bimodal | Normal | Lognormal | Gamma | Cauchy |
| --- | --- | --- | --- | --- | --- |
| *Number of Significant GO:BP Terms* | 0 | 63 | 56 | 187 | NA |
| *Number of Significant GO:BP Terms Unique to Distribution* | 0 | 9 | 1 | 106 | NA |

1. **OV microarray dataset**

|  | Bimodal | Normal | Lognormal | Gamma | Cauchy |
| --- | --- | --- | --- | --- | --- |
| *Number of Significant GO:BP Terms* | 27 | 109 | 113 | 161 | NA |
| *Number of Significant GO:BP Terms Unique to Distribution* | 8 | 31 | 48 | 72 | NA |

1. **AML RNA-seq dataset**

|  | Bimodal | Normal | Lognormal | Gamma | Cauchy |
| --- | --- | --- | --- | --- | --- |
| *Number of Significant GO:BP Terms* | 140 | 88 | 15 | 282 | 43 |
| *Number of Significant GO:BP Terms Unique to Distribution* | 110 | 28 | 0 | 202 | 2 |

1. **GBM RNA-seq dataset**

|  | Bimodal | Normal | Lognormal | Gamma | Cauchy |
| --- | --- | --- | --- | --- | --- |
| *Number of Significant GO:BP Terms* | 82 | 260 | 92 | 135 | 0 |
| *Number of Significant GO:BP Terms Unique to Distribution* | 51 | 165 | 26 | 27 | 0 |

1. **OV RNA-seq dataset**

|  | Bimodal | Normal | Lognormal | Gamma | Cauchy |
| --- | --- | --- | --- | --- | --- |
| *Number of Significant GO:BP Terms* | 82 | 227 | 132 | 227 | NA |
| *Number of Significant GO:BP Terms Unique to Distribution* | 70 | 107 | 41 | 82 | NA |

**Table S11.** List of distribution-specific top five GO:BP terms that are over-represented and unique to each distribution for each patient cohort.

1. **AML microarray dataset**

1. **GBM microarray dataset**

1. **OV microarray dataset**

1. **AML RNA-seq dataset**

1. **GBM RNA-seq dataset**

1. **OV RNA-seq dataset**
